# Inhibition of Iron Uptake Is Responsible for Differential Sensitivity to V-ATPase Inhibitors in Several Cancer Cell Lines

**DOI:** 10.1371/journal.pone.0011629

**Published:** 2010-07-16

**Authors:** Sarah Straud, Iryna Zubovych, Jef K. De Brabander, Michael G. Roth

**Affiliations:** Department of Biochemistry, University of Texas Southwestern Medical Center at Dallas, Dallas, Texas, United States of America; University of Melbourne, Australia

## Abstract

Many cell lines derived from tumors as well as transformed cell lines are far more sensitive to V-ATPase inhibitors than normal counterparts. The molecular mechanisms underlying these differences in sensitivity are not known. Using global gene expression data, we show that the most sensitive responses to HeLa cells to low doses of V-ATPase inhibitors involve genes responsive to decreasing intracellular iron or decreasing cholesterol and that sensitivity to iron uptake is an important determinant of V-ATPase sensitivity in several cancer cell lines. One of the most sensitive cell lines, melanoma derived SK-Mel-5, over-expresses the iron efflux transporter ferroportin and has decreased expression of proteins involved in iron uptake, suggesting that it actively suppresses cytoplasmic iron. SK-Mel-5 cells have increased production of reactive oxygen species and may be seeking to limit additional production of ROS by iron.

## Introduction

Inhibitors of the vacuolar-type (H+)-ATPase (V-ATPase) have been investigated as potential therapeutics for cancer [1], [2] as they show impressive differential cytotoxicity for the 60 cell lines of the NCI COMPARE panel. Additionally, cell lines transformed with oncogenes are more sensitive to V-ATPase inhibitors than are the parental, untransformed cell lines [3], [4]. Many cancer cell lines upregulate expression of V-ATPase subunits compared to normal tissues [1] and V-ATPases are thought to play a role in metastasis [5], [6] and chemoresistance [2], [7]. However, the fundamental mechanisms that determine which cancer cells are most sensitive to V-ATPase inhibitors are currently unknown. This is important knowledge, as inhibiting the V-ATPase itself can inhibit synaptic transmission [8]. Thus proteins involved in cellular processes that are most differentially sensitive to inhibition of the V-ATPase might be better therapeutic targets than the V-ATPase itself.

The V-ATPase is a large, protein complex that can transport protons across membranes against a pH gradient and thus generate the acidic environment found in endocytic organelles, the Golgi apparatus and the Trans-Golgi Network [9]. It is composed of a large, cytosolic hexameric ATPase, V_1_, that is joined by several linkages to an integral membrane complex, V_0_. Hydrolysis of ATP by subunits of V_1_ is converted into mechanical rotation in V_0_ that moves protons from the cytosolic to the lumenal side of the membrane in which V_0_ resides. The activity of the V-ATPase is controlled by multiple mechanisms so that when disassembled, V_1_ does not hydrolyze ATP and V_0_ does not rotate and transport protons [9]. A number of inhibitors of the V-ATPase are known that have distinct binding sites [10].

In both the secretory and the endocytic pathways pH gradients are critical for many functions. The lumen of the endoplasmic reticulum is neutral and that of the Golgi complex is acidic and this difference is used to regulate the binding of escaped ER chaperones in the acidic Golgi by the KDEL receptor, which recycles to release them at the neutral ER [11]. pH decreases across the Golgi complex, so that prohormone convertases are activated at the acidic exit face of the trans-Golgi network and in secretory vesicles, but not earlier in the pathway [12]. In a similar fashion, many lysosomal proenzymes are inactive at the pH of the secretory pathway and are activated after reaching the lysosome, where the pH is usually below 5.0 [13]. In the endocytic pathway, certain ligands, such as low density lipoproteins (LDL), bind receptors at neutral pH at the cell surface and are released when the receptors reach acidic endosomes [14]. In this way LDL is efficiently taken up by the cell and delivers its cargo of cholesterol to lysosomes while the receptor recycles to the cell surface to bind more ligand. Efficient uptake of iron into cells also requires low pH in endosomes. Transferrin, the carrier for extracellular iron, has high affinity for iron and for its cell surface receptor at typical extracellular pH above 7.0. The transferrin receptor is continually internalized and recycles to the plasma membrane, carrying transferrin to acidic endosomes where it releases iron. Iron-free apotransferrin has high affinity for the receptor at low pH and low affinity at neutral pH. Thus, apotransferrin recycles with its receptor back to the plasma membrane where it is released and regains high affinity for extracellular iron [15]. Low pH is also used to establish the identity of endocytic organelles. Certain cytosolic proteins required for regulating membrane traffic bind to the cytoplasmic face of endosome membranes only when the internal pH of the organelle is acidic [16]. Acidification of lysosomes is also required for the process of autophagy [17]. Although normally expressed at low levels at the plasma membrane except in certain acid secreting cells, V-ATPase is over-expressed at the plasma membrane of some cancer cells and may play a role regulating cytosolic pH [5], [6], [18]. Any or all of these essential functions might be more vulnerable to inhibition of the V-ATPase in particular cancer cell backgrounds.

To investigate the basis for the differential sensitivity of cancer cells to inhibitors of the V-ATPase, we have made use of the observation that cells often respond to stress by up-regulating critical components of pathways that are sensed to be failing, as for example the response to the failure of protein folding in the endoplasmic reticulum [19]. Our hypothesis is that a mild inhibition of the V-ATPase will reveal the most sensitive biological functions for that enzyme, which will be indicated by genes that first increase transcription in response to the inhibition. Testing this hypothesis, we have observed that pathways of iron regulation are most sensitive to partial inhibition of the V-ATPase in HeLa cells and that some cancer cell lines most sensitive to V-ATPase inhibitors can be made much more resistant by adding exogenous iron to the culture medium. This suggests that components of iron metabolism or transport may be candidates as therapeutic targets in certain cancers, and these cancers may be identified through their sensitivity to V-ATPase inhibitors or by changed expression of iron transport proteins.

## Results

**Figure 1:**
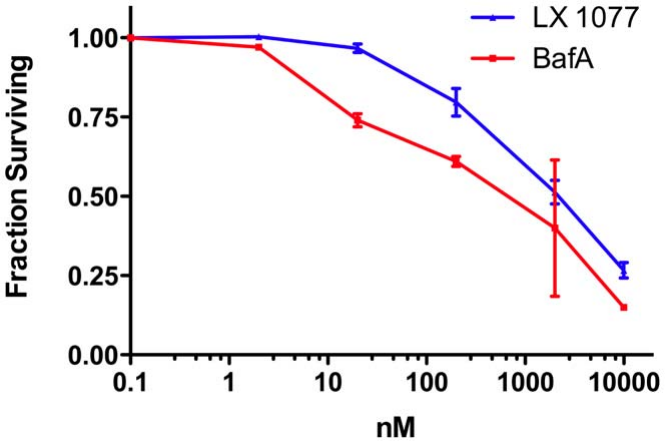
Dose-response of HeLa cells to BafA or LX1077. HeLa cells were cultured in medium containing BafA or LX1077 at the concentrations shown for 48 hours and cell numbers were calculated. Data is normalized to controls treated with 0.1% DMSO. Data are averages of triplicate samples and error bars are SEM.

To determine which metabolic pathways were stressed by low doses of V-ATPase inhibitors, we first determined the dose response to two different inhibitors of the V-ATPase on several cell lines. We wished to find a concentration of each inhibitor that would show an effect on growth, indicating that cells were responding to the stimulus, but which was not so toxic that the response would be prevented or complicated by cells dying during the experiment. The cell lines we tested had IC50s to bafilomycin A that ranged from 10 nM to greater than 10 µM and we chose the moderately sensitive cell line HeLa (IC50 = 150 nM) for our experiments because its relatively shallow dose response made for more uniform growth inhibition experiments, suggesting that the gene expression experiments would also be more reproducible. We also chose to compare two inhibitors of the V-ATPase that act through distinct mechanisms [20], bafilomycin A (Baf) and phenylsalicylihalamide (LX1077), to control for possible off-target effects of each compound. Concentrations of each compound that inhibited cell growth approximately 25% after 48 hours (Figure 1) were chosen for the gene expression experiments. HeLa cells were cultured overnight and then samples were treated with 15 nM Baf for 6, 12, or 24 hours, or 200 nM LX1077 for 12 and 24 hours, and RNA was harvested and processed for analysis by microarray. As controls, samples of HeLa cells treated for the same interval with 0.1% DMSO (the final concentration of the diluent for Baf or LX1077 in experimental samples), were run in parallel with each experimental condition. Each experimental condition was repeated in an independent experiment conducted on a different day.

Two major pathways for uptake of nutrients into cells depend upon the acidity in endosomes supplied by the V-ATPase. Inhibiting the V-ATPase will interfere with the delivery of iron into the cell by preventing the release of iron from transferrin and also prevents uptake of cholesterol by inhibiting the release of LDL from its receptor. Therefore we treated HeLa cells for 6 or 12 hours with deferoxamine (DFO) to chelate iron and separately with medium lacking LDL to mimic inhibiting uptake of exogenous cholesterol through the LDL receptor. Controls were matched samples cultured in normal culture medium. These samples were processed for microarrays exactly as samples treated with V-ATPase inhibitors. In this way we could identify all genes responding by increased transcription when iron was deficient or LDL uptake was blocked. Data was processed by the UT Southwestern DNA Micro Array Core Facility using GCOS software with MAS5 normalization and experimental samples were compared to the DMSO treated samples, or, for DFO or low LDL conditions, samples cultured in normal medium run at the same time to calculate the magnitude of change.

Genes were considered to show significantly increased expression if both samples at one time point with either Baf or LX1077 had a change P value of less than 0.002 and the average of the two samples increased more than 2-fold. In most cases, Baf induced transcription more than LX1077, although the genes responding to Baf also responded to LX1077. The genes that increased transcription earliest are shown in Tables S1, S2 and Table 1. Table S1 presents 47 genes that increased expression when HeLa cells were treated with V-ATPase inhibitors or with low cholesterol medium and represent the response to inhibiting the V-ATPase that is due to blocking delivery of exogenous cholesterol. The response of most of these genes is faster in the presence of the V-ATPase inhibitors than it is to medium lacking LDL, perhaps because medium lacking LDL does not block the delivery of cholesterol from LDL particles that are either bound to their receptor at the cell surface or already released into the endocytic pathway at time the medium deficient in LDL was added. The majority of the genes shown in Table S1 function in lipid biosynthetic pathways and 23 are known targets of SREBP1 or 2 [21]. With the exception of EGLN1 and STARD4, none of the genes that increase transcription early in response to low LDL respond to the iron chelator DFO. EGLN1 encodes an iron-dependent prolylhydroxylase that represses Hif-1α activity under normoxic conditions [22] and is not known to be sensitive to the activity of the cholesterol biosynthetic pathway. However, in yeast and mammals there is cross-talk between cholesterol biosynthesis and pathways of adaptation to hypoxia [23], [24]. STARD4 encodes a cytosolic cholesterol transfer protein that is an SREBP target [21] and also is upregulated by ER stress [25] and might be only indirectly upregulated by the iron chelator. Interestingly, increased transcription of INSIG1, encoding a negative regulator of the SREBP transcription factors that control cholesterol biosynthesis, is one of the faster responses to V-ATPase inhibitors. This is possibly a negative feedback mechanism to limit the transcriptional response once lipid metabolic pathways have increased production of endogenous cholesterol [26].

**Table 1:** Genes Increasing Expression Early with V-ATPase Inhibitors and not with DFO or Low LDL.

			Fold Increase Relative to Control						
Gene	Name	Function or Pathway	Baf 6 hr	Baf 12 hr	LX 12 hr	DFO 6 hr	DFO 12 h	low LDL 6 hr	low LDL 12 hr
CCNG2	cyclin G2	cell cycle control	1.5	2.0	2.0	NC	NC	NC	NC
CLCN6	chloride channel 6, isoform ClC-6a	chloride transport	8.0	2.1	NC	NC	NC	NC	NC
DUSP6	dual specificity phosphatase 6	inactivates ERK2	2.3	2.6	NC	NC	NC	NC	NC
FADSD6	delta-6 fatty acid desaturase	lipid biosynthersis	1.7	3.4	2.4	NC	NC	NC	NC
SREBF2	sterol regulatory element binding transcription factor 2	cholesterol biosynthesis	1.5	2.0	NC	NC	NC	NC	NC
TUBA1A	tubulin, alpha 1a	microtubule protein	1.3	2.1	1.4	NC	NC	NC	NC
WDR37	WD repeat domain 37	unknown	1.9	1.9	NC	NC	NC	NC	NC

Genes upregulated 2-fold or more after 12 hours in cells treated with V-ATPase inhibitors and in cells treated with 100 µM deferoxamine are listed. Baf, 15 nM bafilomycin A; LX, 200 nM LX1077; DFO, 100 µM deferoxamine; low LDL, cells incubated in medium containing LDL depleted serum.

Table S2 lists 64 genes that increased transcription fastest when iron was chelated and also respond to inhibition of the V-ATPase. These are genes in pathways that presumably respond to the inhibition of iron release by transferrin in endosomes when the V-ATPase is inhibited. At least 29 of these genes are known targets of Hif-1α and respond to hypoxia. As one would expect, these genes function in many pathways. Aldolase C is a Hif-1α target [27] and is strongly upregulated by V-ATPase inhibitors. Although we did not detect an early increase in aldolase C in cells treated with low LDL medium, aldolase C is also a known target of SREBPs [21]. Aldolase C binds to the V-ATPase [28] and in yeast is responsible for glucose regulation of V-ATPase activity [29], [30]. Since aldolase C responds to hypoxia in lung epithelial cells and to V-ATPase inhibitors in HeLa cells, whereas the more abundant glycolytic enzyme aldolase A does not, it is possible that a major function of aldolase C in response to lack of iron or hypoxia is to regulate the V-ATPase as part of the mechanism to ensure iron homeostasis. Hif-1α is repressed primarily by being hydroxylated on proline by iron-dependent prolyhydroxylases, which targets it for degradation [31]. By immunoblot we found that incubating HeLa cells in 15 nM Baf for 1 hr stabilized Hif-1α (data not shown), suggesting that pathways regulated by Hif-1α are among the most sensitive to inhibition of the V-ATPase. Thus, V-ATPase inhibitors provide a most effective means for engaging Hif-1α pathways experimentally.

Table 1 presents 7 genes that increased transcription in response to V-ATPase inhibitors, but not in HeLa cells treated with low LDL medium or with the iron chelator. These are candidates for pathways that sense the lack of V-ATPase activity independently of the effect on transferrin or LDL. Other than CLCN6, these genes are not strongly upregulated and several of them are involved in the cell cycle and growth control and may be an early part of the growth inhibition that will occur after longer incubation in the inhibitors. CLCN6 is a chloride channel found in neurons and epithelial cells [32], [33], and localizes to late endosomes [33]. Chloride channels in endosomes work in concert with V-ATPase and thus CLCN6 upregulation may be a response to loss of late endosome function. SREBF2 (SREBP2) is a major regulator of cholesterol biosynthesis and it is primarily regulated post-transcription by membrane traffic and proteolysis [34]. The modest transcriptional increase is likely balanced by the increase in expression of the inhibitor of SREBP activation, INSIG1.

Table 2 presents the results of taking a less restrictive analysis of the genes that change in HeLa cells treated with 15 nM Baf for 6 hr. All genes that showed a statistically significant change (change P value >0.00025) regardless of the direction of change were analyzed with Ingenuity Pathways to determine which canonical pathways in the Ingenuity Database were enriched in genes that were changed in response to Baf. Pathways that responded significantly to Baf contained very few genes that decreased expression, confirming our assumption that the early response would be increased gene expression. By far the most significantly affected pathways were steroid biosynthesis, followed by glycolysis, and involved genes for the most part also identified by our more restrictive analysis.

**Table 2:** Pathways significantly enriched in genes changing expression after 6 h treatment with 15 nM bafilomycin.

Ingenuity Canonical Pathways	-log p value	Ratio	Down	No change or Not present	Up	Molecules
Biosynthesis of Steroids	12.40	0.09	0/128	117/128	11/128	FDPS, MVK, SC5DL, FDFT1, SQLE, EBP, IDI1, DHCR7, HMGCR, LSS, MVD
Glycolysis & Gluconeogenesis	4.10	0.06	0/142	134/142	8/142	GNE, PGM1, ALDOA, ALDOC, PGK1, ENO2, ACSS2, HK2
Fructose & Mannose Metabolism	3.72	0.04	0/145	139/145	6/145	GNE, PFKFB3, ALDOA, ALDOC, PFKFB4, HK2
TR/RXR Activation	3.64	0.07	1/97	90/97	6/97	SLC2A1, SLC16A3, PIK3C2A, LDLR, DIO2, SREBF2, FASN
Inositol Metabolism	3.61	0.07	0/98	91/98	7/98	FADS1, ALDOA, DHCR24, ALDOC, RDH11, SC4MOL, ERO1L
HIF1α Signaling	3.19	0.06	1/108	101/108	6/108	EGLN1, SLC2A1, VEGFA, EGLN3, SLC2A3, PIK3C2A, EDN1
AMPK Signaling	2.56	0.04	0/165	158/165	7/165	PFKFB3, IRS2, PIK3C2A, HMGCR, PFKFB4, AK3L1, FASN
RCC Signaling	2.54	0.07	0/72	67/72	5/72	SLC2A1, VEGFA, EGLN3, MAP2K1, PIK3C2A
Ovarian Cancer Signaling	2.04	0.04	1/135	129/135	5/135	PTGS2, VEGFA, FZD8, MAP2K1, PIK3C2A, EDN1
IGF-1 Signaling	1.99	0.05	1/100	95/100	4/100	GRB10, IRS2, MAP2K1, PIK3C2A, CYR61

Genes changing significantly in duplicate experiments were analyzed with Ingenuity Pathways Analysis software to identify pathways in which a statistically significant number of genes were changed. Only pathways with a –log p value greater than 2 by Fisher's exact T-test are shown. The “ratio” is the fraction of total genes assigned to the pathway that changed expression.

**Figure 2:**
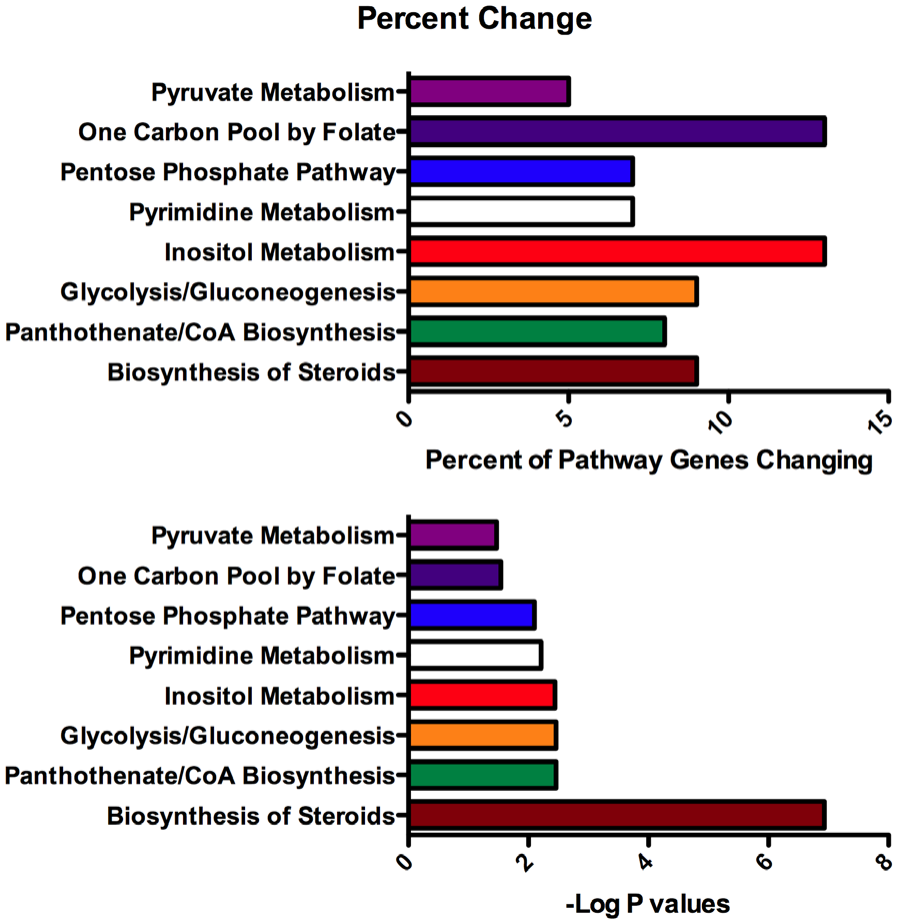
Metabolic functions of genes increasing expression after 24 hours in response to inhibitors of V-ATPase, but not responding to low cholesterol or iron chelation. The genes in Table S4 were analyzed with Ingenuity Pathways to assign them to canonical pathways. The percent of genes in the pathway represented in this data set, and the significance of the change are shown.

After longer intervals of treatment with V-ATPase inhibitors, 52 genes that had increased in response to 12 hours culture in low LDL medium also showed increased expression in cells treated for 24 hours with V-ATPase inhibitors (Table S3). Eight of the cholesterol responsive genes that were upregulated earlier no longer showed an increase and 15 new cholesterol responsive genes showed increased transcription, with 6 of these encoding proteins involved in lipid biosynthetic pathways. 104 “iron responsive” genes (Table S4) were increased in cells treated with V-ATPase inhibitors for 24 hours, including a number of genes encoding glycolytic enzymes or repressors of mitochondrial function, transcription factors and proteins regulating cell growth. STXBP1, which encodes the MUNC-18 regulator of membrane fusion, showed modest increase in response to either depleting cholesterol or iron and a stronger increase in cells treated with V-ATPase inhibitors. This is one of the very few genes encoding proteins that regulate membrane traffic that increased in response to inhibiting the V-ATPase (HIP1R, SV2A and OPTN were the others). Sixty two genes increased expression at least two-fold in response to inhibiting the V-ATPase for 24 hours, but did not respond to cholesterol deficient medium or DFO (Table S5). This set of genes was analyzed using Ingenuity Pathways software to identify metabolic pathways in which genes were significantly upregulated (Figure 2). By far the most significant response was in genes involved in lipid and steroid biosynthesis.

As one would expect, if both increases and decreases in gene expression are considered, after 24 hours in Baf there is a complicated response involving a number of pathways that control growth and response to stress (Table S6). The most significant of these is the NRF2-mediated oxidative stress response, suggesting that inhibiting the V-ATPase increases reactive oxygen species. As important components of the mitochondrial respiration pathway require iron, and inhibition of oxidative phosphorylation can produce ROS, changes in NRF2 mediated pathways could be a secondary consequence of inhibiting iron import from transferrin.

**Figure 3:**
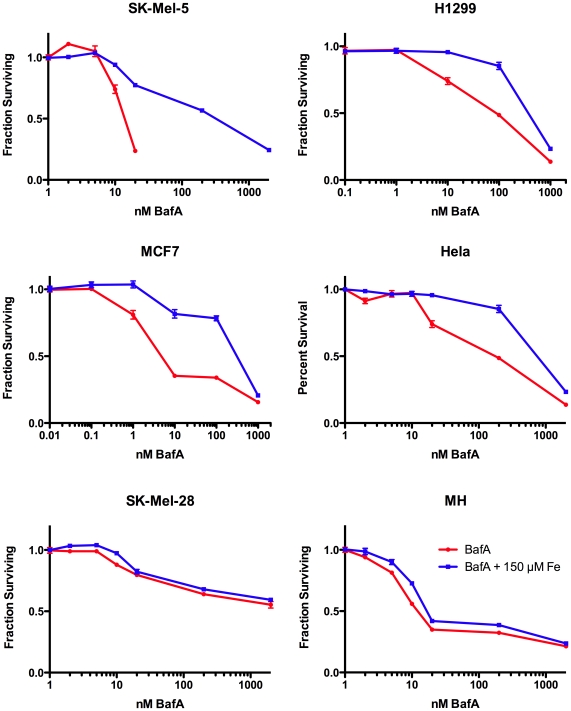
Iron supplement desensitizes cancer cell lines to bafilomycin. The dose-response of six cancer cell lines to Baf in the presence or absence of 150 µM iron citrate was determined as described in the legend to figure 1.

**Figure 4:**
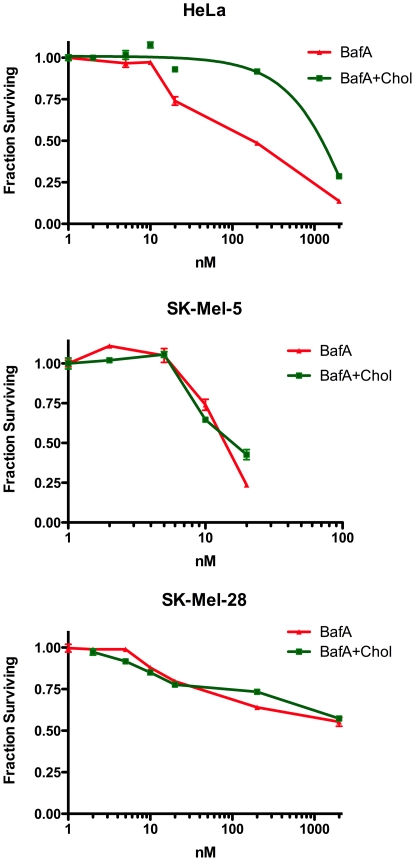
Cholesterol supplement does not desensitize SK-Mel-5 cells to bafilomycin. The dose response curves of HeLa, SK-MeL-5 and SK-Mel-28 cells to Baf in the presence or absence of exogenous cholesterol delivered to cells by methyl-β-cyclodextrin was determined as described in the legend to figure 1.

Our original intent in pursuing these studies was to investigate the causes of differential response to V-ATPase inhibitors seen in cancer cells. Because the major immediate responses to inhibition of the V-ATPase were to the lack of iron or cholesterol, we investigated if these were responsible for the hypersensitivity of some cancer cells to V-ATPase inhibitors (Figure 3). We measured the dose-response of cervical cancer derived HeLa, melanoma derived cell lines SK-Mel-5 and SK-Mel-28, non-small cell lung cancer line H1299, breast cancer MCF-7 and Morris Hepatoma (MH) cells to Baf in the presence or absence of 150 µM iron citrate. Baf had very similar IC50 concentrations for HeLa, MCF7 and H1299. Both MH and SK-Mel-5 cells were 10-fold more sensitive than HeLa, and SK-Mel-28 were highly insensitive (Figure 3). For Hela, SK-Mel-5 and SK-Mel-28 we also tested the effect of adding cholesterol delivered into cells with methy-β-cyclodextrin, which adds cholesterol directly to the plasma membrane [26]. Adding iron to the medium made HeLa cells 20-fold more resistant to growth inhibition caused by 20 to 200 nM bafilomycin, but provided little protection to the cytotoxic effects observed at higher concentrations (Figure 3). Cholesterol was protective for HeLa cells, but not for the Baf-sensitive SK-Mel-5 cells or Baf resistant SK-Mel-28 (Figure 4). This indicates that the cytostatic effects of V-ATPase inhibitors in HeLa cells are largely due to the inhibition of iron and cholesterol uptake, but the cytotoxic effects are due to an alternative, and as yet unknown, function. Supplementing H1299, MCF7 or the more sensitive SK-Mel-5 melanoma cell line with iron protected them at lower concentrations of Baf similar to HeLa cells (Figure 3). In fact, MCF7 or SK-Mel-5 cells treated with iron and bafilomycin had a dose response remarkably similar to HeLa cells treated with bafilomycin alone, suggesting that these cell lines were particularly sensitive to inhibition of iron uptake. Neither iron nor cholesterol had any protective effect on the highly resistant cell line SK-Mel-28 or the quite sensitive MH cell line.

**Figure 5:**
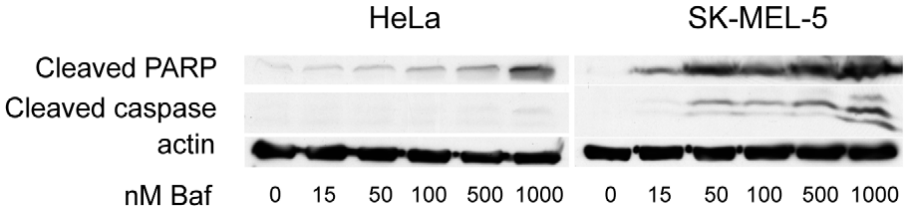
Bafilomycin induces apoptosis. HeLa and SK-Mel-5 cells were treated with indicated concentrations of Baf for 24 hrs and then prepared for immunoblotting with antibodies to cleaved PARP or cleaved caspase 3. The cleaved proteins appear at much lower concentrations of Baf in SK-Mel-5 cells compared to HeLa, with the concentration difference between the appearance of cleaved protein similar to the differences in lethal doses for the two cell lines. The immunoblots shown are representative of two independent experiments.

To investigate the mechanism of cytotoxicity induced by Baf in SK-Mel-5 and HeLa cells, we treated cells of each line with 0 to 1000 nM Baf for 24 hours and then investigated the cleavage of PARP or caspase 3 as signs for the induction of apoptosis (Figure 5). In both cell lines Baf induced apoptosis after 24 hours; however, the concentration required was over 20-fold lower for SK-Mel-5 than HeLa cells, very similar to the differences in lethal doses for the two cell lines. Thus, the cytotoxicity resulting from inhibiting the V-ATPase in the hypersensitive cell line SK-Mel-5 is through induction of apoptosis.

**Figure 6:**
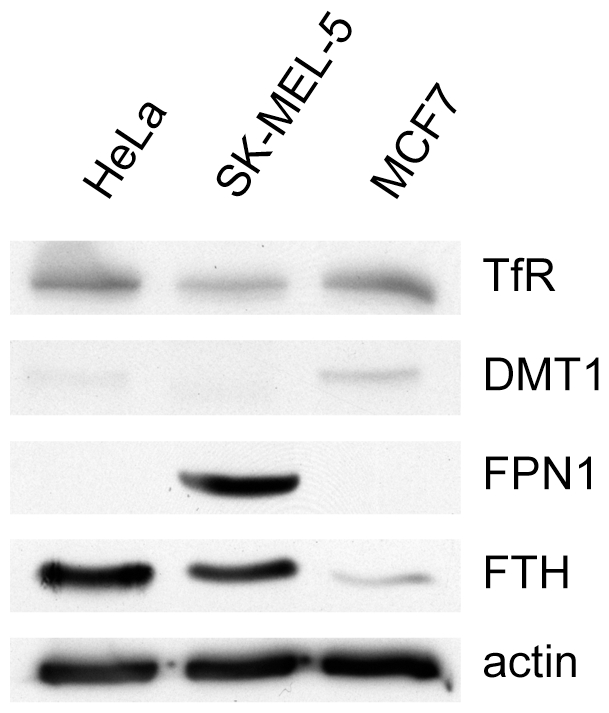
The expression of proteins involved in iron transport in SK-Mel-5 cells indicates a decrease in iron stores. The expression of transferrin receptor 1, ferritin and ferroportin in HeLa and SK-Mel-5 cells in the presence or absence of DFO was measured by immunoblotting. Relative to HeLa cells, SK-Mel-5 have decreased expression of transferrin receptor and ferritin and a large increase in expression of the iron efflux protein ferroportin. TfR, transferrin receptor. DMT1, divalent metal transporter 1. FPN1, ferroportin 1. FTH, ferritin H heavy chain. The immunoblots shown are representative of two independent experiments.

Both SK-Mel-5 cells and MCF7 cells were more sensitive to Baf than HeLa cells and this difference in sensitivity was abolished by supplementing the culture medium with iron (Figure 3). Thus we investigated the expression of proteins involved in iron uptake, storage or efflux in these cell lines and HeLa cells (Figure 6). SK-Mel-5 cells expressed less transferrin receptor than either of the other two cell lines and very low levels of DMT1, the transporter responsible from moving iron across the endosome membrane to the cytoplasm. Remarkably, SK-Mel-5 cells expressed high levels of the iron efflux transporter, ferroportin, which transports iron across the plasma membrane and out of the cell. Ferroportin was not detected in either of the other two cell lines. SK-Mel-5 cells expressed less ferritin than HeLa cells, consistent with having low levels of cytoplasmic iron. Taken together, this pattern of expression suggested that SK-Mel-5 cells were actively maintaining relatively low cytoplasmic iron levels. In contrast, MCF7 cells had levels of transferrin receptor similar to HeLa cells, increased expression of DMT1 and very low levels of the iron storage protein, ferritin. This is a pattern of expression consistent with a cell that has low cytoplasmic iron levels and has upregulated iron transport mechanisms in response. Thus, SK-Mel-5 cells and MCF7 cells, although both hypersensitive to Baf due to inhibition of iron uptake, differed in the molecular mechanisms underlying this vulnerability.

**Figure 7:**
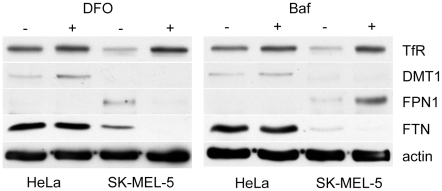
Iron regulation of transferrin receptor, ferroportin and ferritin is intact in SK-Mel-5 cells. Treatment of HeLa or SK-Mel-5 cells with 100 µM DFO for 24 hrs resulted in increased expression of the transferrin receptor in both cell lines, and a decrease in ferroportin and ferritin in SK-Mel-5 cells. Baf treatment had a similar effect, with the exception that ferroportin expression in SK-Mel-5 cells was increased by Baf. HeLa cells did not express ferroportin and SK-Mel-5 cells had very low expression of DMT1. TfR, transferrin receptor. DMT1, divalent metal transporter 1. FPN1, ferroportin 1. FTH, ferritin H heavy chain. The immunoblots shown are representative of two independent experiments.

The differences in expression of transferrin receptor and ferroportin in SK-Mel-5 cells were not due to an inability to regulate these proteins in response to iron. When SK-Mel-5 cells or HeLa cells were treated with DFO to chelate iron, both upregulated expression of transferrin receptor (Figure 7). In response to DFO, transferrin receptor expression in SK-Mel-5 cells was very similar to that in HeLa, indicating that mechanisms for expressing the receptor in response to iron were intact in SK-Mel-5 cells. DFO treatment decreased the expression of ferroportin and ferritin in SK-Mel-5 cells, as one would expect for a cell striving to maintain cellular iron levels. Thus, mechanisms for regulating these proteins by iron were also intact in SK-Mel-5 cells. When SK-Mel-5 cells were treated with Baf, they also increased expression of the transferrin receptor and decreased ferritin. However, ferroportin expression was significantly increased. Since chelating iron decreased ferroportin expression in SK-Mel-5 cells, this response was due to some other effect of inhibiting the V-ATPase.

**Figure 8:**
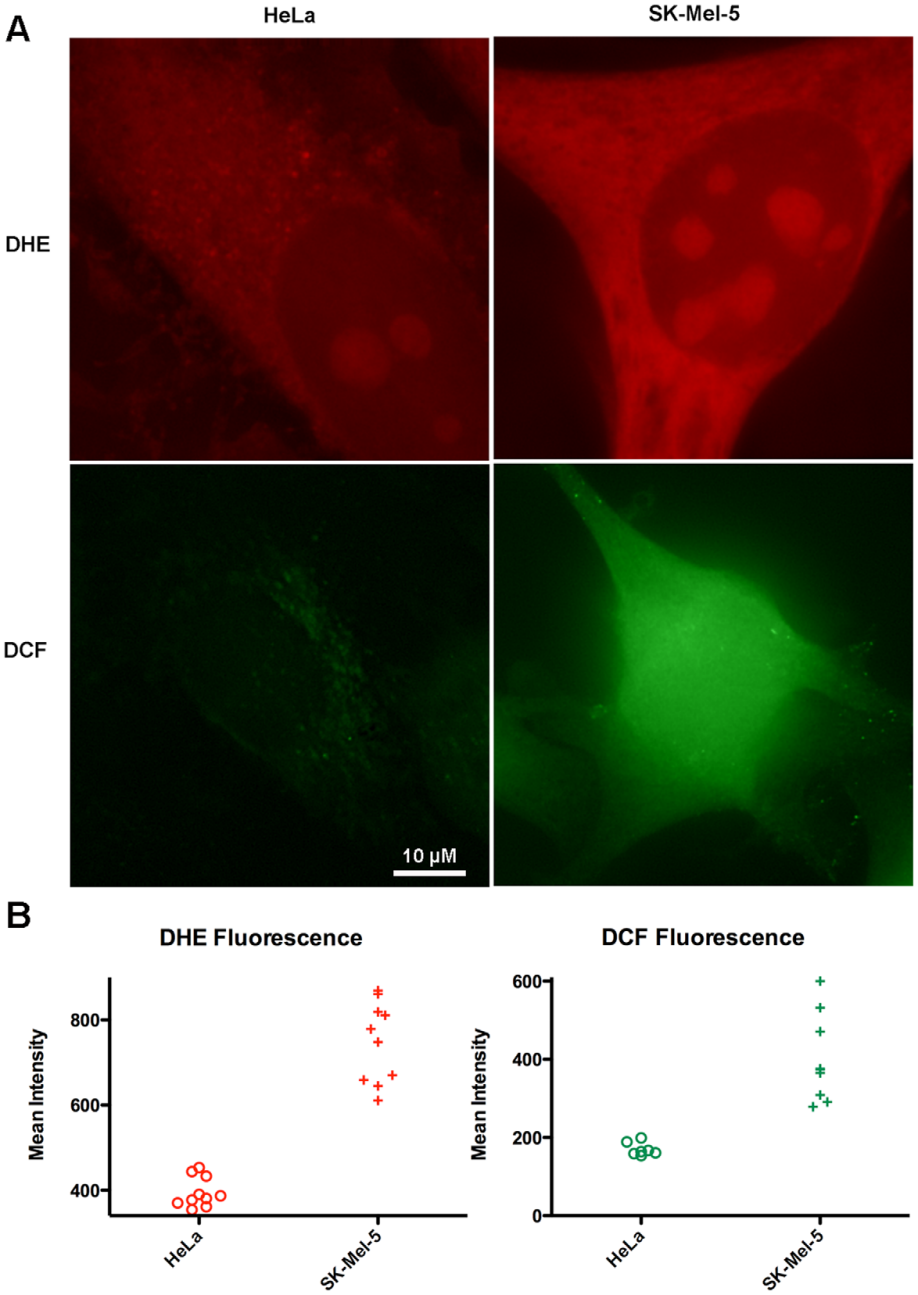
SK-MEL-5 have increased ROS. A. Cells were labeled with the H2O2 sensitive probe DCF, or the superoxide sensitive probe DHE, as described in methods. Cells were photographed with identical camera settings. B. Fluorescent cells were outlined in Image J and the fluorescence within individual cells was measured and graphed with GraphPad Prism.

A possible reason for a cell to actively depress cytoplasmic iron levels would be to protect against the generation of reactive oxygen species (ROS) by iron. To investigate if SK-Mel-5 cells might be producing more ROS, we labeled SK-Mel-5 and HeLa cells with dyes that increase fluorescence in the presence of ROS. Cells were photographed with identical camera settings and fluorescence intensities of cells were measured using Image J software. SK-Mel-5 cells produced significantly more ROS than HeLa cells, measured with both dyes (Figure 8).

**Figure 9:**
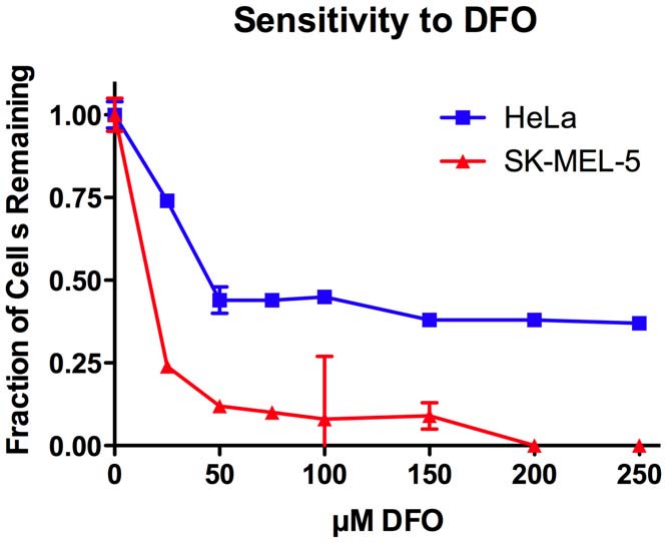
SK-Mel-5 cells are hypersensitive to DFO. Cells were treated with the concentrations of DFO shown as described in Methods and after 72 hours cells were counted. Two wells for each concentration were counted, and the average numbers of two independent experiments were graphed +/− SEM, n = 2.

Since SK-Mel-5 cells appeared to have low levels of intracellular iron and were hypersensitive to Baf, which would further decrease iron uptake, we investigated the sensitivity of SK-Mel-5 cells to the iron chelator DFO (Figure 9). As expected, SK-Mel-5 cells were hypersensitive to DFO compared to HeLa cells. In fact, DFO was only cytostatic for HeLa cells, whereas it was cytotoxic for SK-Mel-5. Adding exogenous iron to the cell culture medium of SK-Mel-5 or HeLa cells had no effect on cell growth (data not shown).

**Figure 10:**
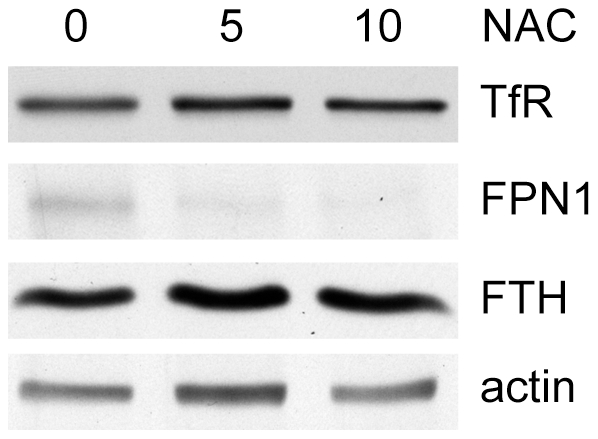
Ferroportin expression in SK-Mel-5 cells is decreased by the antioxidant NAC. SK-Mel-5 cells were treated with NAC as described in the Methods and then processed for immunoblotting with antibodies against the proteins shown. TfR, transferrin receptor. FPN1, ferroportin 1. FTH, ferritin H heavy chain. The immunoblots shown are representative of two independent experiments.

Three observations suggested that ferroportin expression might be induced by ROS in SK-Mel-5 cells. SK-Mel-5 cells had both increased ROS production and increased ferroportin expression. One of the major pathways induced by Baf treatment of HeLa cells for 24 hr was the antioxidant response mediated by NRF2, suggesting that Baf treatment could increase ROS. Baf induced rather than repressed ferroportin expression in SK-Mel-5 cells, whereas lowering iron levels with DFO decreased ferroportin expression. If ferroportin expression was induced by ROS, then treating SK-Mel-5 cells with the anti-oxidant N-acetylcysteine (NAC) would be expected to decrease ferroportin expression. When SK-Mel-5 cells were treated for 48 hrs with increasing concentrations of NAC, ferroportin expression was decreased (Figure 10). Taken together, our data suggest that SK-Mel-5 cells are vulnerable to inhibition of iron uptake because they maintain low iron reserves, perhaps to protect themselves from generating toxic levels of ROS.

## Discussion

Although a number of cell lines derived from tumors or cell lines transformed by oncogenes are highly sensitive to V-ATPase inhibitors, problems of toxicity in animals, particularly neurotoxicity, have prevented development of these inhibitors as anti-cancer agents. The obvious problem is that the V-ATPase is an important enzyme responsible for many fundamental cellular processes and inhibiting it will have many effects in many cell types. This in turn suggests that the hypersensitivity of cancer cells to V-ATPase inhibitors should be due to a vulnerability in one or more of the many processes requiring the V-ATPase and that identifying these vulnerabilities might reveal therapeutic targets that can be exploited with fewer toxic effects on normal tissues. Our data show that inhibition of iron uptake by V-ATPase inhibitors is an important determinant of differential sensitivity of cancer cell lines to these inhibitors, suggesting that mechanisms that control intracellular iron levels might be useful therapeutic targets for certain tumors. Analysis of gene networks upregulated in response to low doses of V-ATPase inhibitors revealed that pathways responsive to decrease in intracellular iron and decrease in exogenous cholesterol were by far the dominant responses, but only iron was related to the hypersensitivity of several cancer cell lines to V-ATPase inhibitors. Our results also indicate that there are multiple mechanisms by which cancer cells are hypersensitive to V-ATPase inhibitors and suggest that measuring the levels of proteins involved in iron uptake, storage and efflux may be useful for identifying tumors that might respond to therapeutics that target iron. Although as rapidly growing cells, one might expect that cancer cells in general might need more iron than normal cells, SK-Mel-5 cells are an example of a cancer-derived cell that appears to be actively limiting intracellular iron through the expression of the efflux transporter, ferroportin. The vulnerability of SK-Mel-5 cells to Baf and the iron chelator DFO is likely to be due to higher than normal production of ROS. Future studies will determine if this is the case and if these higher ROS levels can be manipulated therapeutically. Ferroportin expression might be a particularly good diagnostic for tumors that would be hypersensitive to iron chelators, or to other means of therapeutically increasing ROS.

## Materials and Methods

### Antibodies

The following antibody were used in the study: mouse monoclonal anti – transferrin receptor, cat. # 612124 from BD Biosciences (San Jose, CA), rabbit polyclonal to metal transporter protein (MTP1/IREG1/Ferroportin, cat. # MTP11-A from Alpha Diagnostic International (San Antonio, TX), rabbit polyclonal to divalent cation transporter 1/DMT1/NRAMP2/Slc11a2, cat.# LS-C16687 from LifeSpan Biosciences (Seattle, WA), rabbit polyclonal anti – ferritin FTH1, cat.# 3998, rabbit polyclonal to cleaved PARP (Asp214) cat.# 9541, rabbit polyclonal to cleaved caspase-3 (Asp175), cat.#9661 from Cell Signaling Technology, Inc (Danvers, MA), mouse monoclonal anti-actin, cat.# 69100 from MP Biomedicals (Aurora, Ohio).

### Cell lines

HeLa, SK-MEL-5, SK-MEL-28, and MCF-7 cells were obtained from the American Type Culture Collection. MH were obtained from Ralph Mason (UT Southwestern) and H1993 from John Minna (UT Southwestern). Cells were cultured in DMEM with 10% fetal calf serum in 5% CO2 atmosphere at 37°C.

### Dose-Response to Baf or LX1077 and iron rescue

1×10^4^ cells were added to wells in 96 well plates and the next day a series of concentration of V-ATPase inhibitors or DMSO was added to the wells. After 48 hours the ATP concentration in the wells was measured with CelTiter Glo (Promega). For iron or cholesterol rescue experiments either 150 µM iron citrate (Fe^3+^) or 30 µg/ml cholesterol with 2.5 mM methyl-β-cyclodextrin were included in the medium with the V-ATPase inhibitors.

### MicroArray Experiments

Actively growing HeLa cells were plated in 10 cm diameter dishes in DMEM containing 10% fetal calf serum and grown overnight to just reach confluency. Samples were treated with the following conditions, 15 nM bafilomycin A (Sigma), 2001 nM phenylsalicylihalamide (LX1077), 100 µM deferoxamine mesylate (DFO, Sigma), or DME containing 10% LDL deficient serum (provided by Joseph Goldstein and Michael Brown, UT Southwestern). All samples except the low LDL and matched controls received 0.1% DMSO. Cells were cultured in these conditions for 6, 12, or 24 h and total RNA was isolated with RNEasy Mini Kit (Invitrogen) and at least 2 µg RNA was delivered to the UT Southwestern Microarray Core Facility. Biotinylated cRNA were prepared according to the standard Affymetrix protocol from 2 ug total RNA. Hybridizations to Affymetriz Human Genome U133 Plus 2.0 Arrays and scanning of the arrays with an Affy GeneChip 3000 7G, was conducted according to the manufacturer's instructions. Data was processed with GCOS using MAS5.0 normalization to produce CHP files. The microarray primary data, as CHP and CEL files, has been uploaded to GEO under accession number GSE16870.

### Ingenuity Canonical Pathways Analysis

The functional analysis of pathways enriched in genes which changed expression after 6 or 24 hr treatment with Baf were generated by Ingenuity Pathways Analysis (Ingenuity® Systems, www.ingenuity.com). The software calculates the significance of the association between the data set, in this case all genes with a P change value less than 0.00025, and genes assigned to canonical pathways in the Ingenuity Knowledge Base. This significance is based upon two factors, (1) the fraction of genes in the data set assigned to a pathway compared to all genes assigned to that pathway and (2) a p-value calculated by Fisher's exact test that determines the probability that this association arose by chance alone.

### Western blot analysis

To measure protein levels in response to DFO or Baf, cells were treated with 100 µM DFO or 15 nM Baf for 24 hrs, or left untreated, then washed with 1x PBS and lysed in 150 mM NaCl, 1% NP-40, 0.5% sodium deoxycholate, 0.1% SDS, 50 mM Tris, pH 8 (RIPA buffer). Protein concentrations were determined using DC Protein Assay (Bio-Rad, Hercules, CA) and 30 µg of total protein for each sample was diluted with SDS sample buffer (EMD Chemicals Inc., Gibbstown, NJ), boiled immediately for 15 minutes, and resolved by SDS/PAGE. Proteins were electrotransferred to nitrocellulose membrane (Trans-Blot, Bio-Rad, Hercules, CA) and probed with indicated primary antibody at 4°C, overnight. All antibodies were diluted with 5% Milk in 1x PBS plus 0.5% TWEEN 20. All primary antibodies were used at a dilution of 1∶1,000, with the exception of actin, used at 1∶10,000. Secondary HRP-conjugated goat anti-rabbit and goat anti-mouse antibody (Bio-Rad, Hercules, CA) were used at 1∶3,000 dilution. Protein bands were visualized using the ECL reagent (PerkinElmer Life Sciences, Boston, MA). To measure protein levels, actively growing HeLa, SKMEL5 and MCF7 cells were lysed in RIPA buffer and 30 µg of protein was probed for the protein expression.

To measure apoptosis, HeLa and SK-Mel-5 cells were treated with indicated concentrations of Baf for 24 hrs, then collected, lysed in RIPA buffer and 30 µg of total protein was analyzed by immunoblotting with antibodies to cleaved PARP or cleaved caspase-3.

To measure the effect of N-acetyl-L-cysteine (NAC) on protein expression, SKMEL5 cells were grown in the absence or presence of 5 mM and 10 mM NAC, (Sigma, St. Louis, MO) for 48 hrs. Medium containing NAC was changed every 8 hrs, with freshly dissolved NAC. After 48 hrs treatment, cells were lysed in RIPA buffer and 30 µg of total protein was analyzed by immunoblotting with the antibodies shown. All Western blots shown are representatives of two independent experiments performed on different days with two independent sample sets.

### Intracellular ROS measurements

5-(and-6)-chloromethyl-2′, 7′-dichlorodihydrofluorescein diacetate, acetyl ester (CM-H2DCFDA), cat# C6827 (Invitrogen, Carlsbad, CA) and dihydroethidium (DHE) cat# D23107 (Invitrogen, Carlsbad, CA) are cell permeable ROS-sensitive dyes, that become fluorescent upon oxidation by ROS present in the cytoplasm. The CM-H2DCFDA acts as an H2O2 –sensitive probe, and DHE reacts mainly with superoxide anions [35]. The amount of emitted fluorescence corresponds to the amount of ROS present in the cell. For CM-H2DCFDA the excitation and emission wavelengths were 488 nm and 530 nm, respectively, for DHE the excitation and emission wavelengths were 518 nm and 605 nm, respectively. HeLa and SK-Mel-5 cells were grown on glass coverslips in 12 well plates for 2 days prior the experiment, then 25 µM of CM-H2DCFDA or 5 µM of DHA, freshly diluted in the cell culture media, was added and plates returned back to incubator for 2 hrs, for incubation at 37°C in the dark. After incubation, cells were washed in PBS and fixed in 3.7% buffered paraformaldehyde for 10 minutes, then washed with PBS and mounted on glass slides with Prolong Gold mounting medium with DAPI (Invitrogen, Carlsbad, CA). Cells were examined with a DeltaVision deconvolution microscope and all images collected with the same camera settings. Fluorescence intensity was measure using Image J software and graphed with GraphPad Prism.

### DFO sensitivity

To measure sensitivity to DFO, approximately 250,000 HeLa or SK-Mel-5 cells were plated per well in 12-well tissue culture plates. The next day, cells were treated with indicated dosages of DFO, and 72 hrs later cell numbers were determined by using a Beckman Cell Counter (Coulter, Miami, FL). Two wells for each concentration were counted, and the average numbers of two independent experiments were graphed +/− SEM, n = 2.

## Supporting Information

Table S1 Genes Increasing Expression Early with V-ATPase Inhibitors and with Low LDL. Genes upregulated 2-fold or more after 12 hours in cells treated with V-ATPase inhibitors and in cells incubated in medium lacking LDL are listed. Baf, 15 nM bafilomycin A; LX, 200 nM LX1077; DFO, 100 µM deferoxamine; low LDL, cells incubated in medium containing LDL depleted serum.
(0.12 MB DOC)

Table S2 Genes Increasing Expression Early with V-ATPase Inhibitors and with DFO. Genes upregulated 2-fold or more after 12 hours in cells treated with V-ATPase inhibitors and in cells treated with 100 µM deferoxamine are listed. Baf, 15 nM bafilomycin A; LX, 200 nM LX1077; DFO, 100 µM deferoxamine; low LDL, cells incubated in medium containing LDL depleted serum.
(0.13 MB DOC)

Table S3 Genes Increasing Expression Late with V-ATPase Inhibitors and with Low LDL. Genes upregulated 2-fold or more after 24 hours in cells treated with V-ATPase inhibitors and in cells incubated in medium lacking LDL are listed. Baf, 15 nM bafilomycin A; LX, 200 nM LX1077; DFO, 100 µM deferoxamine; low LDL, cells incubated in medium containing LDL depleted serum.
(0.12 MB DOC)

Table S4 Genes Increasing Expression Late with V-ATPase Inhibitors and with DFO. Genes upregulated 2-fold or more after 24 hours in cells treated with V-ATPase inhibitors and in cells treated with 100 µM deferoxamine are listed in order of increase with Baf for 24 hours. Baf, 15 nM bafilomycin A; LX, 200 nM LX1077; DFO, 100 µM deferoxamine; low LDL, cells incubated in medium containing LDL depleted serum.
(0.19 MB DOC)

Table S5 Genes Increasing Expression Late with V-ATPase Inhibitors and not with DFO or Low LDL. Genes upregulated 2-fold or more after 24 hours in cells treated with V-ATPase inhibitors and in cells treated with 100 µM deferoxamine are listed in order of increase with Baf at 24 hours. Baf, 15 nM bafilomycin A; LX, 200 nM LX1077; DFO, 100 µM deferoxamine; low LDL, cells incubated in medium containing LDL depleted serum.
(0.12 MB DOC)

Table S6 Pathways significantly enriched in genes changing expression after 24 h treatment with 15 nM bafilomycin. Genes changing significantly in duplicate experiments were analyzed with Ingenuity Pathways Analysis software to identify pathways in which a statistically significant number of genes were changed. Only pathways with a -log p value greater than 2 by Fisher's exact T-test are shown. The “ratio” is the fraction of total genes assigned to the pathway that changed expression.
(0.09 MB DOC)

## References

[1] Lebreton S, Jaunbergs J, Roth MG, Ferguson DA, De Brabander JK (2008). Evaluating the potential of vacuolar ATPase inhibitors as anticancer agents and multigram synthesis of the potent salicylihalamide analog saliphenylhalamide.. Bioorg Med Chem Lett.

[2] Sasazawa Y, Futamura Y, Tashiro E, Imoto M (2009). Vacuolar H(+)-ATPase inhibitors overcome Bcl-xL-mediated chemoresistance through restoration of a caspase-independent apoptotic pathway.. Cancer Sci.

[3] Boyd MR, Farina C, Belfiore P, Gagliardi S, Kim JW (2001). Discovery of a novel antitumor benzolactone enamide class that selectively inhibits mammalian vacuolar-type (H+)-atpases.. J Pharmacol Exp Ther.

[4] Kim JW, Shin-ya K, Furihata K, Hayakawa Y, Seto H (1999). Oximidines I and II: Novel Antitumor Macrolides from Pseudomonas sp.. The Journal of Organic Chemistry.

[5] Hinton A, Sennoune SR, Bond S, Fang M, Reuveni M (2009). Function of a Subunit Isoforms of the V-ATPase in pH Homeostasis and in Vitro Invasion of MDA-MB231 Human Breast Cancer Cells.. J Biol Chem.

[6] Sennoune SR, Bakunts K, Martinez GM, Chua-Tuan JL, Kebir Y (2004). Vacuolar H+-ATPase in human breast cancer cells with distinct metastatic potential: distribution and functional activity.. Am J Physiol Cell Physiol.

[7] You H, Jin J, Shu H, Yu B, Milito AD (2009). Small interfering RNA targeting the subunit ATP6L of proton pump V-ATPase overcomes chemoresistance of breast cancer cells.. Cancer Lett.

[8] Porciuncula LO, Rocha JB, Tavares RG, Ghisleni G, Reis M (2003). Methylmercury inhibits glutamate uptake by synaptic vesicles from rat brain.. Neuroreport.

[9] Forgac M (2007). Vacuolar ATPases: rotary proton pumps in physiology and pathophysiology.. Nat Rev Mol Cell Biol.

[10] Bowman EJ, Bowman BJ (2005). V-ATPases as drug targets.. J Bioenerg Biomembr.

[11] Scheel AA, Pelham HRB (1996). Purification and Characterization of the Human KDEL Receptor.. Biochemistry.

[12] RouillÈ Y, Duguay SJ, Lund K, Furuta M, Gong Q (1995). Proteolytic Processing Mechanisms in the Biosynthesis of Neuroendocrine Peptides: The Subtilisin-like Proprotein Convertases.. Frontiers in Neuroendocrinology.

[13] Saroussi S, Nelson N (2009). Vacuolar H(+)-ATPase-an enzyme for all seasons.. Pflugers Arch.

[14] Davis CG, Goldstein JL, Sudhof TC, Anderson RG, Russell DW (1987). Acid-dependent ligand dissociation and recycling of LDL receptor mediated by growth factor homology region.. Nature.

[15] Dautry-Varsat A, Ciechanover A, Lodish HF (1983). pH and the recycling of transferrin during receptor-mediated endocytosis.. Proc Natl Acad Sci U S A.

[16] Gu F, Gruenberg J (2000). ARF1 regulates pH-dependent COP functions in the early endocytic pathway.. J Biol Chem.

[17] Klionsky DJ, Elazar Z, Seglen PO, Rubinsztein DC (2008). Does bafilomycin A1 block the fusion of autophagosomes with lysosomes?. Autophagy.

[18] Sennoune SR, Luo D, Martinez-Zaguilan R (2004). Plasmalemmal vacuolar-type H+-ATPase in cancer biology.. Cell Biochem Biophys.

[19] Lin JH, Walter P, Yen TS (2008). Endoplasmic reticulum stress in disease pathogenesis.. Annu Rev Pathol.

[20] Xie XS, Padron D, Liao X, Wang J, Roth MG (2004). Salicylihalamide A inhibits the V0 sector of the V-ATPase through a mechanism distinct from bafilomycin A1.. J Biol Chem.

[21] Horton JD, Shah NA, Warrington JA, Anderson NN, Park SW (2003). Combined analysis of oligonucleotide microarray data from transgenic and knockout mice identifies direct SREBP target genes.. Proc Natl Acad Sci U S A.

[22] Bruick RK, McKnight SL (2002). Transcription. Oxygen sensing gets a second wind.. Science.

[23] Hughes AL, Todd BL, Espenshade PJ (2005). SREBP pathway responds to sterols and functions as an oxygen sensor in fission yeast.. Cell.

[24] Nguyen AD, McDonald JG, Bruick RK, DeBose-Boyd RA (2007). Hypoxia stimulates degradation of 3-hydroxy-3-methylglutaryl-coenzyme A reductase through accumulation of lanosterol and hypoxia-inducible factor-mediated induction of insigs.. J Biol Chem.

[25] Yamada S, Yamaguchi T, Hosoda A, Iwawaki T, Kohno K (2006). Regulation of human STARD4 gene expression under endoplasmic reticulum stress.. Biochem Biophys Res Commun.

[26] Brown AJ, Sun L, Feramisco JD, Brown MS, Goldstein JL (2002). Cholesterol addition to ER membranes alters conformation of SCAP, the SREBP escort protein that regulates cholesterol metabolism.. Mol Cell.

[27] Jean JC, Rich CB, Joyce-Brady M (2006). Hypoxia results in an HIF-1-dependent induction of brain-specific aldolase C in lung epithelial cells.. Am J Physiol Lung Cell Mol Physiol.

[28] Lu M, Holliday LS, Zhang L, Dunn WA, Gluck SL (2001). Interaction between aldolase and vacuolar H+-ATPase: evidence for direct coupling of glycolysis to the ATP-hydrolyzing proton pump.. J Biol Chem.

[29] Lu M, Ammar D, Ives H, Albrecht F, Gluck SL (2007). Physical interaction between aldolase and vacuolar H+-ATPase is essential for the assembly and activity of the proton pump.. J Biol Chem.

[30] Lu X, Qin W, Li J, Tan N, Pan D (2005). The growth and metastasis of human hepatocellular carcinoma xenografts are inhibited by small interfering RNA targeting to the subunit ATP6L of proton pump.. Cancer Res.

[31] Semenza GL (2009). Regulation of cancer cell metabolism by hypoxia-inducible factor 1.. Semin Cancer Biol.

[32] Kida Y, Uchida S, Miyazaki H, Sasaki S, Marumo F (2001). Localization of mouse CLC-6 and CLC-7 mRNA and their functional complementation of yeast CLC gene mutant.. Histochem Cell Biol.

[33] Poet M, Kornak U, Schweizer M, Zdebik AA, Scheel O (2006). Lysosomal storage disease upon disruption of the neuronal chloride transport protein ClC-6.. Proc Natl Acad Sci U S A.

[34] Brown MS, Goldstein JL (1997). The SREBP pathway: regulation of cholesterol metabolism by proteolysis of a membrane-bound transcription factor.. Cell.

[35] Owusu-Ansah E, Yavari A, Mandal S, Banerjee U (2008). Distinct mitochondrial retrograde signals control the G1-S cell cycle checkpoint.. Nat Genet.

