# Supplementary material for: Inhibition of Iron Uptake Is Responsible for Differential Sensitivity to V-ATPase Inhibitors in Several Cancer Cell Lines
Source: PLoS One. 2010 Jul 16;5(7):e11629. doi: 10.1371/journal.pone.0011629 (PMC2905441; doi:10.1371/journal.pone.0011629)
Supplement: Table S1 — Genes Increasing Expression Early with V-ATPase Inhibitors and with Low LDL. Genes upregulated 2-fold or more after 12 hours in cells treated with V-ATPase inhibitors and in cells incubated in medium lacking LDL are listed. Baf, 15 nM bafilomycin A; LX, 200 nM LX1077; DFO, 100 µM deferoxamine; low LDL, cells incubated in medium containing LDL depleted serum. (0.12 MB DOC) [file pone.0011629.s001.doc]

|  |  |  | **Fold Increase Relative to Control** | | | | | | |
| --- | --- | --- | --- | --- | --- | --- | --- | --- | --- |
| **Gene** | **Name** | **Function or Pathway** | **Baf 6 hr** | **Baf 12 hr** | **LX 12 hr** | **DFO 6 hr** | **DFO 12h** | **low LDL 6 hr** | **low LDL 12 hr** |
|  | **Lipid Metabolism** |  |  |  |  |  |  |  |  |
| ACAT2 | acetyl-Coenzyme A acetyltransferase 2 | lipid metabolism | 1.9 | 5.7 | 2.5 | NC | NC | NC | 7.4 |
| ACSS2 | acyl-CoA synthetase short-chain family member 2 | lipid metabolism | 1.9 | 6.5 | 2.7 | NC | NC | NC | 8.6 |
| C14orf1 | chromosome 14 open reading frame 1 | sterol biosynthesis | 1.7 | 4.4 | 2.4 | NC | NC | NC | 7.2 |
| CYP51A1 | lanosterol 14-alpha demethylase | cholesterol biosynthesis | 2.1 | 4.6 | 2.6 | NC | NC | NC | 7.5 |
| DHCR24 | 24-dehydrocholesterol reductase | cholesterol biosynthesis | 2.1 | 3.1 | 2.0 | NC | NC | NC | 7.0 |
| DHCR7 | 7-dehydrocholesterol reductase | cholesterol biosynthesis | 2.1 | 5.1 | 2.5 | NC | NC | 1.2 | 7.2 |
| EBP | emopamil binding protein (sterol isomerase) | cholesterol biosynthesis | 1.4 | 2.9 | 1.7 | NC | NC | 1.3 | 7.2 |
| FADS1 | fatty acid desaturase 1 | phospholipid biosynthesis | 1.9 | 3.0 | 2.1 | NC | NC | 1.3 | 6.8 |
| FASN | fatty acid synthase | fatty acid synthesis | 1.7 | 2.1 | 1.6 | NC | NC | NC | 7.2 |
| FDFT1 | farnesyl-diphosphate farnesyltransferase 1 | cholesterol biosynthesis | 2.8 | 6.7 | 3.2 | NC | NC | NC | 8.6 |
| FDPS | farnesyl diphosphate synthase | cholesterol biosynthesis | 3.5 | 8.3 | 2.8 | NC | NC | 2.0 | 7.8 |
| HMGCR | 3-hydroxy-3-methylglutaryl-Coenzyme A reductase | cholesterol biosynthesis | 3.5 | 5.7 | 3.0 | NC | NC | 2.1 | 8.2 |
| HMGCS1 | 3-hydroxy-3-methylglutaryl-Coenzyme A synthase 1 | cholesterol biosynthesis | 4.3 | 14.9 | 3.7 | NC | NC | NC | 7.6 |
| HSD17B7 | hydroxysteroid (17-beta) dehydrogenase 7 | sterol biosynthesis | 2.0 | 4.6 | 2.5 | NC | NC | NC | 7.7 |
| IDI1 | isopentenyl-diphosphate delta isomerase | cholesterol biosynthesis | 4.1 | 8.6 | 4.1 | NC | NC | 2.5 | 9.4 |
| INSIG1 | insulin induced gene 1 | cholesterol biosynthesis | 5.1 | 6.7 | 5.1 | NC | NC | NC | 8.3 |
| LDLR | low density lipoprotein receptor | cholesterol uptake | 3.1 | 4.4 | 2.6 | NC | NC | 2.8 | 8.2 |
| LPIN1 | phosphatidate phosphatase | phospholipid biosynthesis | 3.2 | 8.9 | 2.6 | NC | NC | 2.5 | 8.5 |
| LSS | lanosterol synthase (2,3-oxidosqualene-lanosterol cyclase) | sterol biosynthesis | 1.9 | 4.0 | 2.5 | NC | NC | 1.2 | 7.8 |
| MVK | mevalonate kinase | cholesterol biosynthesis | 4.6 | 8.6 | 3.9 | NC | NC | NC | 8.6 |
| NPC1 | Niemann-Pick disease, type C1 | cholesterol transport | 1.5 | 2.1 | 1.7 | NC | NC | 1.3 | 6.8 |
| NSDHL | NAD(P) dependent steroid dehydrogenase-like | cholesterol biosynthesis | 1.8 | 4.1 | 2.3 | NC | NC | NC | 7.6 |
| PCSK9 | proprotein convertase subtilisin/kexin type 9 | cholesterol metabolism | 2.4 | 4.1 | 2.5 | NC | NC | NC | 7.9 |
| NAMPT | nicotinamide phosphoribosyltransferase | biosynthesis of NAD | 1.4 | 2.1 | 1.5 | NC | NC | NC | 6.6 |
| PNPLA3 | patatin-like phospholipase domain containing 3 | triacylglycerol hydrolysis | 1.7 | 2.1 | 1.6 | NC | NC | NC | 6.7 |
| RDH11 | retinol dehydrogenase 11 (all-trans and 9-cis) | retinol metabolism | 1.6 | 2.2 | 1.5 | NC | NC | NC | 6.6 |
| SC4MOL | sterol-C4-methyl oxidase-like | sterol biosynthesis | 3.9 | 7.5 | 4.1 | NC | NC | 2.9 | 8.7 |
| SC5DL | sterol-C5-desaturase (ERG3 delta-5-desaturase homolog, fungal)-like | cholesterol biosynthesis | 1.6 | 2.4 | 1.7 | NC | NC | 1.4 | 7.1 |
| SCD | stearoyl-CoA desaturase (delta-9-desaturase) | fatty acid synthesis | 2.3 | 3.2 | 2.0 | NC | NC | 1.5 | 8.2 |
| SQLE | squalene epoxidase | sterol biosynthesis | 3.9 | 4.6 | 2.8 | NC | NC | 2.0 | 7.7 |
| STARD4 | START domain containing 4 | cholesterol transport | 3.7 | 5.9 | 3.1 | 1.4 | 1.6 | 1.8 | 7.6 |
|  |  |  |  |  |  |  |  |  |  |
|  | **Glucose Metabolism** |  |  |  |  |  |  |  |  |
| SLC2A3P1 | solute carrier family 2 (facilitated glucose transporter), member 3 pseudogene 1 | unknown | 1.7 | 2.0 | 1.6 | NC | NC | NC | 6.8 |
| SLC2A6 | solute carrier family 2 (facilitated glucose transporter), member 6 | hexose transport | 2.2 | 7.7 | 2.0 | NC | NC | NC | 7.4 |
|  |  |  |  |  |  |  |  |  |  |
|  | **Lysosome Related** |  |  |  |  |  |  |  |  |
| NEU1 | lysosomal sialidase | lysosome sialic acid glycosidase | 1.5 | 3.1 | 2.6 | NC | NC | NC | 6.7 |
| GNE | glucosamine (UDP-N-acetyl)-2-epimerase/N-acetylmannosamine kinase | biosynthesis of N-acetylsialic acid | 1.5 | 2.1 | 1.5 | NC | NC | 1.4 | 6.9 |
| WIPI1 | WD repeat domain, phosphoinositide interacting 1 | may function in autophagy | 1.4 | 2.1 | 1.6 | NC | NC | 1.3 | 6.8 |
|  |  |  |  |  |  |  |  |  |  |
|  | **Intercellular Signaling** |  |  |  |  |  |  |  |  |
| TNFSF9 | tumor necrosis factor (ligand) superfamily, member 9 | cytokine | 1.9 | 2.5 | 2.0 | NC | NC | 1.4 | 6.8 |
| VASH2 | vasohibin 2 | angiogenesis inhibitor | 1.6 | 3.0 | 2.1 | NC | NC | NC | 7.0 |
|  |  |  |  |  |  |  |  |  |  |
|  | **Iron Metabolism** |  |  |  |  |  |  |  |  |
| EGLN1 | HIF-prolyl hydroxylase 1 | hypoxia response | 2.2 | 2.8 | 1.7 | 4.4 | 5.5 | NC | 2.55 |
| FLVCR1 | feline leukemia virus subgroup C cellular receptor | heme transporter | 1.7 | 2.2 | NC | NC | NC | NC | 6.7 |
|  |  |  |  |  |  |  |  |  |  |
|  | **Miscellaneous** |  |  |  |  |  |  |  |  |
| MICA | MHC class I polypeptide-related sequence A | stress-induced antigen | 1.4 | 2.8 | 2.2 | NC | NC | NC | 6.5 |
| IER3 | immediate early response 3 | cell proliferation/apoptosis | 1.4 | 3.1 | 1.7 | NC | NC | NC | 6.6 |
| KLF11 | Kruppel-like factor 11 | transcription repressor | 1.6 | 2.5 | 1.6 | NC | NC | NC | 6.7 |
| YPEL5 | yippee-like 5 | unknown | 1.4 | 2.8 | 2.4 | NC | NC | 1.6 | 6.8 |
| KLHL24 | kelch-like 24 | unknown | 2.3 | 2.9 | 2.8 | NC | NC | 4.0 | NC |
| MAGEA4 | melanoma antigen family A, 4 | unknown | 2.0 | 4.1 | 2.4 | NC | NC | NC | 8.0 |
| TMEM97 | transmembrane protein 97 | unknown | 1.9 | 4.0 | 2.0 | NC | NC | NC | 7.8 |
