# Supplementary material for: Inhibition of Iron Uptake Is Responsible for Differential Sensitivity to V-ATPase Inhibitors in Several Cancer Cell Lines
Source: PLoS One. 2010 Jul 16;5(7):e11629. doi: 10.1371/journal.pone.0011629 (PMC2905441; doi:10.1371/journal.pone.0011629)
Supplement: Table S2 — Genes Increasing Expression Early with V-ATPase Inhibitors and with DFO. Genes upregulated 2-fold or more after 12 hours in cells treated with V-ATPase inhibitors and in cells treated with 100 µM deferoxamine are listed. Baf, 15 nM bafilomycin A; LX, 200 nM LX1077; DFO, 100 µM deferoxamine; low LDL, cells incubated in medium containing LDL depleted serum. (0.13 MB DOC) [file pone.0011629.s002.doc]

|  |  |  | **Fold Increase Relative to Control** | | | | | | |
| --- | --- | --- | --- | --- | --- | --- | --- | --- | --- |
| **Gene** | **Name** | **Function or Pathway** | **Baf 6 hr** | **Baf 12 hr** | **LX 12 hr** | **DFO 6 hr** | **DFO 12h** | **low LDL 6 hr** | **low LDL 12 hr** |
|  | **Hif-1a Targets** |  |  |  |  |  |  |  |  |
| ADM | adrenomedullin | hypotensive peptide precursor | 2.8 | 3.0 | 2.4 | 7.5 | 6.3 | NC | NC |
| AK3 | adenylate kinase 3 | nucleotide, nucleic acid metabolism | 2.5 | 3.0 | 1.8 | 5.5 | 5.3 | NC | NC |
| ALDOC | aldolase C | glycolysis | 3.9 | 13.9 | 4.3 | 3.5 | 11.3 | NC | NC |
| BHLHE40 | basic helix-loop-helix family, member e40 | hypoxia induced | 3.5 | 8.0 | 2.4 | 2.8 | 4.3 | NC | NC |
| BNIP3 | BCL2adenovirus E1B interacting protein 3 | transcription factor | 2.6 | 4.3 | 2.8 | 5.3 | 7.2 | NC | NC |
| BNIP3L | BCL2/adenovirus E1B interacting protein 3-like | transcription factor | 2.2 | 3.6 | 2.5 | 4.3 | 6.3 | NC | NC |
| CA9 | carbonic anhydrase IX | Hif1a responsive | 2.8 | 7.7 | 3.5 | 8.3 | 16.6 | NC | NC |
| CCNG2 | cyclin G2 | cell cycle control | 2.2 | 3.4 | 2.1 | 2.5 | 5.1 | 1.6 | NC |
| EGLN1 | HIF-prolyl hydroxylase 1 | hypoxia response | 2.2 | 2.8 | 1.7 | 4.4 | 5.5 | NC | 2.55 |
| EGLN3 | HIF-prolyl hydroxylase 3 | hypoxia response | 2.3 | 1.9 | NC | 9.5 | 7.2 | NC | NC |
| ENO2 | enolase 2 | glycolysis | 7.2 | 8.0 | 5.5 | 11.3 | 43.7 | NC | NC |
| ERO1L | ERO1-like | hypoxia response | 1.5 | 2.0 | NC | 2.8 | 3.4 | NC | NC |
| ERRFI1 | ERBB receptor feedback inhibitor 1 | hypoxia induced | 1.3 | 2.2 | 1.8 | 3.7 | 5.5 | NC | NC |
| FAM162A | HGTD-P | Hif1 alpha target - proapoptotic | 1.4 | 2.1 | 1.4 | 2.9 | 4.6 | NC | NC |
| HIG2 | hypoxia-inducible protein 2 | hypoxia response | 2.0 | 2.5 | 1.5 | 8.3 | 8.9 | NC | NC |
| HK2 | hexokinase 2 | glycolysis | 2.3 | 2.8 | 1.6 | 4.4 | 4.4 | NC | NC |
| INSIG2 | insulin induced protein 2 | cholesterol biosynthesis | 2.3 | 3.4 | 1.9 | 5.3 | 5.9 | NC | NC |
| JMJD1A | jumonji domain containing 1A | histone demethylase | 2.4 | 2.6 | 2.1 | 3.1 | 2.8 | NC | NC |
| MXI1 | MAX interactor 1 | Myc antagonist | 1.9 | 2.1 | 1.4 | 5.3 | 4.8 | 1.4 | NC |
| NDRG1 | N-myc downstream regulated | cell differentiation / stress inducible | 5.7 | 6.5 | 4.1 | 12.1 | 17.8 | NC | NC |
| P4HA1 | procollagen-proline, 2-oxoglutarate 4-dioxygenase | colloagen prolylhydroxylase | 1.8 | 2.7 | 1.8 | 2.8 | 4.4 | NC | NC |
| PDK1 | pyruvate dehydrogenase kinase, isoenzyme 1 | glucose metabolism | 3.0 | 4.9 | 3.0 | 5.5 | 7.5 | NC | NC |
| PFKFB4 | 6-phosphofructo-2-kinase/fructose-2,6-biphosphatase 4 | fructose 2,6-bisphosphate metabolism | 1.7 | 2.9 | 1.7 | 3.7 | 2.9 | NC | NC |
| PTGS2 | prostaglandin-endoperoxide synthase 2 | prostaglandin biosynthesis | 2.0 | 3.5 | 2.2 | 3.1 | 3.2 | NC | NC |
| SLC2A3 | GLUT3 | glucose transport | 4.3 | 8.0 | 2.8 | 8.0 | 5.3 | NC | NC |
| TMEM45A | transmembrane protein 45A | unknown | 2.4 | 3.6 | 2.3 | 3.6 | 6.1 | NC | NC |
| VEGF | vascular endothelial growth factor | growth factor | 4.1 | 4.9 | 2.4 | 10.2 | 4.9 | NC | NC |
| VLDLR | very low density lipoprotein receptor | lipid transport | 2.1 | 3.5 | 3.1 | 4.4 | 7.7 | NC | NC |
| WSB1 | WD repeat and SOCS box-containing 1 | E3 ubiquitin ligase | 1.6 | 2.0 | 1.5 | 3.0 | 3.2 | NC | NC |
|  |  |  |  |  |  |  |  |  |  |
|  | **Glucose Metabolism** |  |  |  |  |  |  |  |  |
| SLC1A1 | solute carrier family 1 member 1 | high affinity glutamate transporter | 1.6 | 2.1 | 1.4 | 2.4 | 2.5 | NC | NC |
| SLC2A1 | solute carrier family 2 (Glut1) | glucose transport | 1.7 | 1.9 | 1.4 | 3.4 | 2.9 | NC | NC |
|  |  |  |  |  |  |  |  |  |  |
|  | **Cell Proliferation** |  |  |  |  |  |  |  |  |
| BTG1 | B-cell translocation gene 1 | negative regulator of cell cycle | 1.5 | 2.0 | 1.4 | 1.8 | 2.1 | 1.2 | NC |
| EFNA3 | ephrin-A3 | Eph receptor ligand | 4.3 | 7.2 | NC | 5.7 | 9.2 | NC | NC |
| GDF15 | TGFB1 superfamily member | growth factor | 2.3 | 4.3 | 3.2 | 2.0 | 2.9 | NC | NC |
| RASSF7 | RalGDS/AF-6) domain family 7 | required for mitosis | 29.9 | 19.0 | 26.0 | 30.9 | 36.8 | NC | NC |
| OSMR | oncostatin M receptor | cell proliferation | 1.4 | 2.0 | 1.3 | 2.9 | 4.4 | NC | NC |
| PTPRR | protein tyrosine phosphatase, receptor type R | regulator of MAP kinases | 1.9 | 2.8 | 2.7 | 1.5 | 1.9 | NC | NC |
|  |  |  |  |  |  |  |  |  |  |
|  | **Miscellaneous** |  |  |  |  |  |  |  |  |
| AKAP13 | A kinase (PRKA) anchor protein 13 | Rho GEF | 2.2 | 1.7 | NC | 2.5 | 2.1 | NC | NC |
| CYB5B | cytochrome b5 type B (outer mitochondrial membrane) | electron transport | 1.3 | 2.1 | 1.5 | 1.6 | 2.5 | NC | NC |
| FUT11 | fucosyltransferase 1 | protein glycosylation | 2.3 | 3.0 | 2.2 | 6.1 | 6.1 | NC | NC |
| KCTD11 | potassium channel tetramerisation domain containing 11 | potassium ion transport | 2.5 | 4.3 | NC | 8.3 | 8.0 | NC | NC |
| LRP2BP | low density lipoprotein receptor-related protein | scaffold protein | 2.8 | 3.6 | 2.5 | 6.3 | 5.7 | NC | NC |
| MMAB | methylmalonic aciduria (cobalamin deficiency) cblB type | adenosylcobalamin synthesis | 1.4 | 2.0 | 1.4 | 1.9 | 1.8 | NC | NC |
| ORAI3 | ORAI calcium release-activated calcium modulator 3 | calcium channel component | 1.9 | 2.6 | 2.5 | 2.0 | 2.6 | NC | NC |
| TMEM47 | transmembrane protein 47 | cell-cell adhesion | 1.7 | 2.1 | 1.6 | 2.1 | 2.2 | NC | NC |
| TPBG | oncofetal trophoblast glycoprotein | cell adhesion | 1.6 | 2.5 | 1.6 | 2.4 | 2.3 | NC | NC |
| UFSP2 | UFM1-specific peptidase 2 | processes ubiquitin fold modifiers | 2.8 | 3.6 | 2.5 | 6.3 | 5.7 | NC | NC |
| USP53 | ubiquitin specific peptidase 53 | ubiquitin peptidase | 1.5 | 2.3 | 1.7 | 2.5 | 3.6 | NC | NC |
| TSC22D3 | TSC22 domain family, member 3 | transcriptional regulator | 1.9 | 2.8 | 1.7 | 1.4 | 1.9 | NC | NC |
| MAFF | v-maf musculoaponeurotic fibrosarcoma oncogene homolog F | transcription factor | 2.6 | 4.4 | 2.3 | 6.7 | 6.3 | NC | NC |
| ZBTB1 | zinc finger and BTB domain containing 1 | transcription factor | 3.7 | 2.2 | 1.9 | 2.5 | 3.1 | NC | NC |
| ZNF292 | zinc finger protein 292 | transcription factor | 1.9 | 1.9 | 1.6 | 1.9 | 2.5 | NC | NC |
| ANKZF1 | ankyrin repeat and zinc finger domain containing 1 | unknown | 1.5 | 2.4 | NC | 3.9 | 2.5 | NC | NC |
| C3orf58 | chromosome 3 open reading frame 58 | unknown | 1.6 | 2.0 | 1.4 | 2.6 | 2.9 | NC | NC |
| C4orf3 | HCV F-transactivated protein 1 | unknown | 1.3 | 2.1 | NC | 2.6 | 3.1 | NC | NC |
| C7orf60 | hypothetical protein FLJ31818 | unknown | 2.1 | 1.7 | NC | 2.2 | 2.9 | NC | NC |
| MASTL | microtubule associated serine/threonine kinase-like | unknown | 1.3 | 2.2 | NC | 3.7 | 5.5 | NC | NC |
| SCAND2 | SCAN domain containing 2 | unknown | 2.5 | 2.5 | NC | 4.0 | 5.3 | NC | NC |
| TM7SF2 | transmembrane 7 superfamily member 2 | unknown | NC | 7.7 | 4.4 | 2.4 | 1.5 | NC | NC |
| SOX9 | SRY (sex determining region Y)-box 9 | transcription regulator | 1.5 | 2.1 | NC | 1.7 | 1.6 | NC | NC |
