# Supplementary material for: Inhibition of Iron Uptake Is Responsible for Differential Sensitivity to V-ATPase Inhibitors in Several Cancer Cell Lines
Source: PLoS One. 2010 Jul 16;5(7):e11629. doi: 10.1371/journal.pone.0011629 (PMC2905441; doi:10.1371/journal.pone.0011629)
Supplement: Table S3 — Genes Increasing Expression Late with V-ATPase Inhibitors and with Low LDL. Genes upregulated 2-fold or more after 24 hours in cells treated with V-ATPase inhibitors and in cells incubated in medium lacking LDL are listed. Baf, 15 nM bafilomycin A; LX, 200 nM LX1077; DFO, 100 µM deferoxamine; low LDL, cells incubated in medium containing LDL depleted serum. (0.12 MB DOC) [file pone.0011629.s003.doc]

|  |  |  | **Average Fold Increase** | | | | | |
| --- | --- | --- | --- | --- | --- | --- | --- | --- |
| **Gene** | **Name** | **Function or Pathway** | **Baf 12h** | **LX 12h** | **Baf 24h** | **LX 24 h** | **DFO 12h** | **Low LDL 12h** |
|  | **Lipid Metabolism** |  |  |  |  |  |  |  |
| ACAT2 | acetyl-Coenzyme A acetyltransferase 2 | lipid biosynthesis | 5.7 | 2.5 | 8.6 | 4.8 | NC | 2.30 |
| ACSS2 | acyl-CoA synthetase short-chain family member | lipid biosynthesis | 6.5 | 2.7 | 15.5 | 5.7 | NC | 3.48 |
| C14orf1 | chromosome 14 open reading frame 1 | ERG28 homolog, sterol synthesis | 4.4 | 2.4 | 7.5 | 4.8 | NC | 2.14 |
| CYP51A1 | cytochrome P450 family 51, subfamily A, polypeptide 1 | cholesterol biosynthesis | 2.9 | 1.9 | 3.1 | 2.5 | NC | 2.14 |
| CYP51P2 | cytochrome P450, subfamily 51 pseudogene 2 | cholesterol biosynthesis | 4.6 | 2.6 | 5.9 | 3.6 | NC | 2.38 |
| DHCR24 | 24-dehydrocholesterol reductase | cholesterol biosynthesis | 3.1 | 2.0 | 4.3 | 2.5 | NC | 1.87 |
| DHCR7 | 7-dehydrocholesterol reductase | cholesterol biosynthesis | 5.1 | 2.5 | 7.7 | 4.3 | NC | 2.14 |
| EBP | emopamil-binding protein (sterol isomerase) | cholesterol biosynthesis | 2.6 | 1.7 | 5.9 | 3.2 | NC | 1.93 |
| FABP3 | fatty acid binding protein 3 | transport of long-chain fatty acids | 2.4 | NC | 29.9 | 5.7 | NC | 1.74 |
| FASN | fatty acid synthase | fatty acid synthesis | 2.1 | 1.6 | 3.6 | 2.4 | NC | 2.07 |
| FDFT1 | farnesyl-diphosphate farnesyltransferase 1 | cholesterol biosynthesis | 3.7 | 2.5 | 3.9 | 3.0 | NC | 2.64 |
| FDPS | farnesyl diphosphate synthase | cholesterol biosynthesis | 8.3 | 2.8 | 8.3 | 3.6 | NC | 2.73 |
| HMGCR | 3-hydroxy-3-methylglutaryl-Coenzyme A reductase | cholesterol biosynthesis | 7.2 | 3.1 | 6.5 | 4.1 | NC | 2.93 |
| HMGCS1 | 3-hydroxy-3-methylglutaryl-Coenzyme A synthase 1 | cholesterol biosynthesis | 14.9 | 3.7 | 8.0 | 2.5 | NC | 2.55 |
| HSD17B7 | hydroxysteroid (17-beta) dehydrogenase 7 | sterol biosynthesis | 4.6 | 2.5 | 5.1 | 2.5 | NC | 2.64 |
| IDI1 | isopentenyl-diphosphate delta isomerase | cholesterol biosynthesis | 8.9 | 3.9 | 8.0 | 5.7 | NC | 4.29 |
| INSIG1 | insulin induced gene 1 | cholesterol biosynthesis | 6.1 | 4.6 | 7.0 | 5.7 | NC | 3.14 |
| LDLR | low density lipoprotein receptor | cholesterol uptake | 4.4 | 2.6 | 5.1 | 3.1 | NC | 3.14 |
| LPIN1 | phosphatidate phosphatase | phospholipid biosynthesis | 8.9 | 2.6 | 9.2 | 3.7 | NC | 3.36 |
| MVK | mevalonate kinase | isoprenoid and sterol synthesis | 8.6 | 3.9 | 13.0 | 13.5 | NC | 3.48 |
| NPC1 | Niemann-Pick disease, type C1 | cholesterol transport | 2.1 | 1.7 | 4.1 | 2.3 | NC | 1.74 |
| NSDHL | NAD(P) dependent steroid dehydrogenase-like | cholesterol biosynthesis | 4.1 | 2.3 | 5.1 | 3.4 | NC | 2.46 |
| PANK3 | pantothenate kinase 3 | biosynthesis of CoA | 2.0 | 1.6 | 2.5 | 1.7 | NC | 1.68 |
| PCSK9 | proprotein convertase subtilisin/kexin type 9 | cholesterol metabolism | 4.1 | 2.5 | 9.2 | 4.1 | NC | 2.83 |
| PCYT2 | phosphate cytidylyltransferase 2, ethanolamine | lipid biosynthesis | 1.6 | 1.8 | 2.9 | 2.2 | NC | 1.87 |
| PNPLA3 | patatin-like phospholipase domain containing 3 | triacylglycerol lipase | 2.1 | 1.6 | 3.4 | 2.0 | NC | 1.62 |
| SC4MOL | sterol-C4-methyl oxidase-like | cholesterol biosynthesis | 7.5 | 4.1 | 7.0 | 5.3 | NC | 3.61 |
| SC5DL | sterol-C5-desaturase | cholesterol biosynthesis | 2.4 | 1.7 | 3.4 | 2.3 | NC | 2.00 |
| SCD | stearoyl-CoA desaturase (delta-9-desaturase) | fatty acid synthesis | 3.2 | 2.0 | 2.6 | 2.2 | NC | 3.14 |
| SQLE | squalene epoxidase | sterol biosynthesis | 4.6 | 2.8 | 5.5 | 3.6 | NC | 2.64 |
|  |  |  |  |  |  |  |  |  |
|  | **Iron Metabolism** |  |  |  |  |  |  |  |
| EGLN1 | egl nine homolog 1 | prolyl hydroxylase | 5.9 | 3.1 | 6.3 | 3.1 | 1.62 | 2.55 |
| FLVCR | feline leukemia virus subgroup C cellular receptor | heme transporter | 2.3 | 1.5 | 2.8 | NC | NC | 1.80 |
|  |  |  |  |  |  |  |  |  |
|  | **Lysosome Function** |  |  |  |  |  |  |  |
| GNE | glucosamine (UDP-N-acetyl)-2-epimerase/N-acetylmannosamine kinase | biosynthesis of N-acetylneuraminic acid | 2.1 | 1.5 | 2.5 | NC | NC | 1.80 |
| NEU1 | lysosomal sialidase | lysosome function | 3.1 | 2.6 | 7.0 | 4.8 | NC | 1.62 |
| STXBP1 | syntaxin binding protein 1 | MUNC-18 vesicle fusion | 2.4 | 1.9 | 3.5 | 2.5 | 1.52 | 1.68 |
|  |  |  |  |  |  |  |  |  |
|  | **Miscellaneous** |  |  |  |  |  |  |  |
| ARID5B | AT rich interactive domain 5B (MRF1-like) | transcriptional repressor | 1.5 | 1.5 | 2.7 | 2.0 | NC | 1.41 |
| JUND | jun D proto-oncogene | antiapoptotic | 1.9 | NC | 2.1 | 1.7 | NC | 1.74 |
| KLF11 | Kruppel-like factor 11 | transcription repressor | 2.5 | 1.6 | 2.3 | NC | NC | 1.62 |
| DNAJB9 | DnaJ (Hsp40) homolog, subfamily B, member 9 | Chaperon | 1.9 | NC | 2.1 | 1.5 | NC | 1.68 |
| MICA | MHC class I polypeptide-related sequence A | stress-induced antigen | 2.1 | NC | 2.6 | 1.8 | NC | 1.41 |
| NUPR1 | nuclear protein 1 | stress-induced protein | 1.9 | 1.9 | 3.7 | 3.5 | NC | 1.87 |
| MMAB | cob(I)alamin adenosyltransferase | coenzyme for methylmalonyl-CoA mutase | 2.8 | 2.2 | 4.1 | 3.7 | NC | 1.41 |
| QPRT | quinolinate phosphoribosyltransferase | NAD pyrophosphorylase | 1.9 | 1.4 | 3.1 | 2.1 | NC | 1.46 |
| PRKCDBP | protein kinase C, delta binding protein | possible growth suppressor | 1.9 | NC | 2.5 | 1.9 | NC | 1.52 |
| PRUNE2 | prune homolog 2 | negative regulator of RhoA | NC | 1.7 | 4.1 | 3.2 | NC | 1.57 |
| LOC644051 | isocitrate dehydrogenase pseudogene | hypothetical protein | 2.2 | 1.6 | 2.1 | 1.9 | NC | 1.68 |
| LZTFL1 | leucine zipper transcription factor-like 1 | unknown | 1.7 | 1.6 | 2.1 | 1.4 | NC | 1.57 |
| MAGEA4 | melanoma antigen family A, 4 | unknown function | 4.1 | 2.4 | 4.8 | 3.0 | NC | 2.93 |
| TMEM97 | transmembrane protein 97 | unknown | 4.4 | 2.4 | 11.3 | 3.9 | NC | 2.83 |
| TNFSF9 | tumor necrosis factor (ligand) superfamily,member 9 | T cell cytokine | 2.5 | 2.0 | 3.9 | 2.5 | NC | 1.68 |
| VASH2 | vasohibin 2 | antiangiogenic activity | 3.0 | 2.1 | 3.7 | 2.1 | NC | 1.93 |
