# Supplementary material for: Inhibition of Iron Uptake Is Responsible for Differential Sensitivity to V-ATPase Inhibitors in Several Cancer Cell Lines
Source: PLoS One. 2010 Jul 16;5(7):e11629. doi: 10.1371/journal.pone.0011629 (PMC2905441; doi:10.1371/journal.pone.0011629)
Supplement: Table S4 — Genes Increasing Expression Late with V-ATPase Inhibitors and with DFO. Genes upregulated 2-fold or more after 24 hours in cells treated with V-ATPase inhibitors and in cells treated with 100 µM deferoxamine are listed in order of increase with Baf for 24 hours. Baf, 15 nM bafilomycin A; LX, 200 nM LX1077; DFO, 100 µM deferoxamine; low LDL, cells incubated in medium containing LDL depleted serum. (0.19 MB DOC) [file pone.0011629.s004.doc]

|  |  |  | **Average Fold Increase** | | | | | |
| --- | --- | --- | --- | --- | --- | --- | --- | --- |
| **Gene** | **Name** | **Function or Pathway** | **Baf 12h** | **LX 12h** | **Baf 24h** | **LX 24h** | **DFO 12h** | **Low LDL 12h** |
| ALDOC | aldolase C | glycolysis | 13.9 | 4.3 | 57.7 | 17.1 | 11.3 | NC |
| CA9 | carbonic anhydrase IX | Hif1a responsive | 7.7 | 3.5 | 28.8 | 12.1 | 16.6 | NC |
| RASSF7 | RalGDS/AF-6) domain family 7 | required for mitosis | 19.0 | 26.0 | 23.4 | 26.0 | 36.8 | NC |
| SPAG4 | sperm associated antigen 4 | dense fiber binding protein | 6.7 | 3.1 | 10.9 | 3.0 | 5.7 | NC |
| NDRG1 | N-myc downstream regulated gene 1 | cell differentiation,  stress inducible | 6.5 | 4.1 | 10.6 | 5.5 | 17.8 | NC |
| ENO2 | enolase 2 | glycolysis | 8.0 | 5.5 | 10.2 | 5.1 | 43.7 | NC |
| VLDLR | very low density lipoprotein receptor | cholesterol uptake | 3.5 | 3.1 | 9.8 | 4.0 | 7.7 | NC |
| SLC2A3 | solute carrier family 2 (GLUT3) | glucose uptake | 5.7 | 2.4 | 8.9 | 2.6 | 4.4 | NC |
| GDF15 | TGFB1 superfamily member | growth factor | 4.3 | 3.2 | 8.9 | 3.7 | 2.9 | NC |
| MAFF | v-maf musculoaponeurotic fibrosarcoma oncogene homolog F | transcription factor | 4.4 | 2.3 | 8.9 | 2.7 | 6.3 | NC |
| PTPRR | protein tyrosine phosphatase, receptor type R | associates with **β**4 adaptin, inhibits ERK2 | 2.5 | 3.0 | 6.3 | 4.1 | 2.5 | NC |
| BNIP3 | BCL2/adenovirus E1B 19kDa interacting protein 3 | Hif1a induced transcription factor | 4.6 | 2.6 | 6.3 | 3.9 | 9.5 | NC |
| EGLN1 | egl nine homolog 1 | hypoxia response | 5.9 | 3.1 | 6.3 | 3.1 | 1.6 | 2.5 |
| ADM | adrenomedullin | hypotensive peptide | 3.0 | 2.4 | 6.1 | 2.3 | 6.3 | NC |
| CCL28 | chemokine (C-C motif) ligand 28 | T cell chemokine | NC | 6.7 | 5.9 | NC | 6.7 | NC |
| INSIG2 | insulin induced protein 2 | cholesterol biosynthesis | 3.4 | 1.9 | 5.5 | 2.2 | 5.9 | NC |
| TMEM45A | transmembrane protein 45A | unknown | 3.6 | 2.3 | 5.3 | 3.5 | 6.1 | NC |
| PDK1 | pyruvate dehydrogenase kinase, isoenzyme 1 | inactivates PDH | 4.9 | 3.0 | 5.1 | 3.7 | 7.5 | NC |
| CCNG2 | cyclin G2 | cell cycle regulation | 3.4 | 2.1 | 4.9 | 2.8 | 5.1 | NC |
| RAB20 | RAB20, member RAS oncogene family | membrane traffic | 2.2 | NC | 4.6 | 1.7 | 4.3 | NC |
| ORAI3 | ORAI calcium release-activated calcium modulator 3 | calcium channel component | 2.6 | 2.5 | 4.3 | 3.2 | 2.6 | NC |
| FUT11 | fucosyltransferase 11 | protein glycosylation | 3.0 | 1.8 | 4.3 | 2.0 | 5.3 | NC |
| P4HA2 | procollagen-proline, 2-oxoglutarate 4-dioxygenase alpha polypeptide II | ER prolyl hydroxylase | 2.0 | 1.4 | 4.0 | 1.9 | 3.6 | NC |
| CXCL1 | chemokine (C-X-C motif) ligand 1 | GRO1 oncogene | 5.5 | 2.9 | 4.0 | 2.6 | 2.8 | NC |
| VEGFC | vascular endothelial growth factor C | growth factor | 3.9 | 2.5 | 4.0 | 2.7 | 5.3 | NC |
| HIG2 | hypoxia-inducible protein 2 | hypoxia response | 2.5 | 1.5 | 4.0 | 1.5 | 8.9 | NC |
| BNIP3L | BCL2adenovirus E1B 19kD-interacting protein 3-like | proapoptotic | 4.8 | 2.9 | 4.0 | 2.7 | 6.3 | NC |
| SCAND2 | SCAN domain containing 2 | unknown | 2.5 | NC | 4.0 | 1.7 | 5.3 | NC |
| BHLHB2 | basic helix-loop-helix domain containing, class B, 2 | DEC1 transcription factor | 8.0 | 2.4 | 3.9 | NC | 4.3 | NC |
| ARL10 | ADP-ribosylation factor-like 10 | unknown | 1.7 | NC | 3.9 | 1.7 | 3.4 | NC |
| VEGF | vascular endothelial growth factor | growth factor | 4.9 | 2.4 | 3.7 | 2.7 | 4.9 | NC |
| PTGS2 | prostaglandin-endoperoxide synthase 2 | prostaglandin synthesis | 3.5 | 2.2 | 3.7 | 2.4 | 3.2 | NC |
| CUGBP2 | CUG triplet repeat, RNA binding protein 2 | regulates splicing | 2.4 | NC | 3.7 | 1.5 | 2.5 | NC |
| PFKFB4 | 6-phosphofructo-2-kinase/fructose-2,6-biphosphatase 4 | fructose 2,6-bisphosphate metabolism | 2.9 | 1.7 | 3.6 | 1.8 | 2.9 | NC |
| ANKRD37 | ankyrin repeat domain 37 | low density lipoprotein receptor-related protein binding protein | 3.6 | 2.5 | 3.6 | 2.1 | 5.7 | NC |
| AK3L1 | adenylate kinase 3-like 1 | nucleotide & nucleic acid metabolism | 3.1 | 2.0 | 3.6 | 2.0 | 4.8 | NC |
| P4HA1 | procollagen-proline, 2-oxoglutarate4-dioxygenase | ER prolyl hydroxylase | 2.7 | 1.8 | 3.5 | 2.6 | 4.4 | NC |
| IER3 | immediate early response 3 | Erk substrate, | 2.1 | NC | 3.5 | 1.6 | 3.1 | NC |
| STXBP1 | syntaxin binding protein 1 | vesicle fusion | 2.4 | 1.9 | 3.5 | 2.5 | 1.5 | 1.7 |
| LOXL2 | lysyl oxidase-like 2 | crosslinks elastin | 1.9 | NC | 3.4 | 1.5 | 4.3 | NC |
| C4orf3 | chromosome 4 open reading frame 3 | HCV F-transactivated protein 1 | 2.3 | 1.7 | 3.4 | 2.3 | 3.1 | NC |
| ZNF395 | zinc finger protein 395 | transcription factor | 2.5 | NC | 3.2 | 2.0 | 5.9 | NC |
| PDK3 | pyruvate dehydrogenase kinase, isoenzyme 3 | inactivates PDH | 2.3 | NC | 3.1 | 1.8 | 4.6 | NC |
| CNOT3 | CCR4-NOT transcription complex, subunit 3 | transcription regulation | 1.9 | NC | 3.1 | 2.4 | 4.0 | NC |
| OSMR | oncostatin M receptor | cytokine receptor | 2.0 | 1.5 | 3.0 | 1.9 | 2.4 | NC |
| KDM3A | lysine (K)-specific demethylase 3A | histone demethylase | 2.6 | 2.1 | 3.0 | 2.1 | 2.8 | NC |
| PPP1R3B | protein phosphatase 1, regulatory (inhibitor) subunit 3B | putative phosphatase regulator | NC | 1.9 | 3.0 | 1.6 | 3.7 | NC |
| SMAD9 | SMAD family member 9 | signal transduction | 1.6 | 1.5 | 3.0 | 1.4 | 2.6 | NC |
| TSC22D3 | TSC22 domain family, member 3 | transcription regulation | 2.8 | 1.7 | 3.0 | 2.2 | 1.9 | NC |
| TMCC1 | transmembrane and coiled-coil domain family 1 | unknown | NC | NC | 3.0 | 1.6 | 2.0 | NC |
| HK2 | hexokinase 2 | glycolysis | 2.8 | 1.6 | 2.9 | 1.9 | 4.4 | NC |
| ERO1L | ERO1-like | protein disulfide isomerase like | 2.1 | NC | 2.9 | 1.6 | 3.4 | NC |
| HEATR1 | HEAT repeat containing 1 | rRNA processing | 1.9 | 1.6 | 2.9 | 1.4 | 2.4 | NC |
| TPBG | trophoblast glycoprotein | cell adhesion | 2.5 | 1.6 | 2.8 | 1.9 | 2.3 | NC |
| TMEM47 | transmembrane protein 47 | PMP22/EMP/claudin protein family | 2.1 | 1.6 | 2.8 | 1.8 | 2.2 | NC |
| FAM26F | family with sequence similarity 26, member F | predicted protein, function unknown | 1.9 | NC | 2.8 | 1.7 | 3.0 | NC |
| CDA | cytidine deaminase | pyrimidine salvaging | NC | NC | 2.8 | 1.8 | 1.5 | NC |
| LOC391552 | hypothetical | unknown | 1.4 | 1.5 | 2.8 | 2.0 | 1.8 | NC |
| DPYSL2 | dihydropyrimidinase-like 2 | dihydropyrimidinase | 2.1 | NC | 2.7 | 1.8 | 1.6 | NC |
| SLC1A1 | solute carrier family 1 member 1 | high affinity glutamate transporter | 2.1 | NC | 2.7 | 1.6 | 2.5 | NC |
| MPP1 | membrane protein, palmitoylated 1 | MAGUK | 1.8 | 1.4 | 2.7 | 1.7 | 2.2 | NC |
| CCDC107 | coiled-coil domain containing 107 | unknown | 1.7 | NC | 2.7 | 1.6 | 1.9 | NC |
| TNIP1 | TNFAIP3 interacting protein 1 | ERK interacting protein | 1.9 | 1.5 | 2.6 | 1.9 | 1.7 | NC |
| STC2 | stanniocalcin 2 | Hif-1 responsive, growth suppressor | 1.6 | NC | 2.6 | 1.5 | 1.9 | NC |
| C3orf28 | HGTD-P | Hif1a responsive | 2.1 | NC | 2.6 | 1.7 | 4.6 | NC |
| EGLN3 | egl nine homolog 3 | hypoxia response, prolylhydroxylase | 1.9 | NC | 2.6 | 1.6 | 7.2 | NC |
| DUSP5 | dual specificity phosphatase 5 | inactivates Erk1 | 1.7 | NC | 2.6 | 1.5 | 1.9 | NC |
| NAMPTL | nicotinamide phosphoribosyltransferase-like | unknown | 2.4 | NC | 2.6 | 1.6 | 3.1 | NC |
| FZD7 | frizzled homolog 7 | Wnt receptor signaling pathway | 1.8 | NC | 2.6 | 1.6 | 1.5 | NC |
| ITGA2 | integrin, alpha 2 (CD49B, alpha 2 subunit of VLA-2 receptor) | cell adhesion and signaling | 2.0 | 1.4 | 2.5 | 1.6 | 1.5 | NC |
| MXI1 | MAX interactor 1 | inhibits myc | 2.1 | NC | 2.5 | 1.5 | 4.8 | NC |
| CITED2 | Cbp/p300-interacting transactivator | transcription factor in hypoxia response | 3.4 | 2.0 | 2.5 | 1.8 | 6.7 | NC |
| WSB1 | WD repeat and SOCS box-containing 1 | ubiquitin ligase E3 | 1.8 | NC | 2.5 | 1.5 | 2.7 | NC |
| AK3P1 | adenylate kinase 3 pseudogene 1 | unknown | 2.5 | 1.6 | 2.5 | 1.8 | 3.9 | NC |
| BTG1 | B-cell translocation gene 1 | negative regulator of cell cycle | 2.0 | NC | 2.5 | 1.6 | 2.1 | NC |
| SNAI2 | snail homolog 2 | transcription factor | NC | 1.5 | 2.5 | NC | 1.8 | NC |
| PBEF1 | pre-B-cell colony enhancing factor 1 | cytokine | 2.0 | NC | 2.4 | 1.6 | 2.2 | NC |
| GYS1 | glycogen synthase 1 | glycogen synthesis | 1.8 | NC | 2.4 | 1.3 | 2.9 | NC |
| PRRX1 | paired related homeobox 1 | represses MAF | 1.6 | NC | 2.4 | 1.4 | 1.8 | NC |
| TP53INP1 | tumor protein p53 inducible nuclear protein 1 | stress induced, proapoptotic | 2.1 | 1.9 | 2.4 | 1.7 | 1.8 | NC |
| SOX9 | SRY (sex determining region Y)-box 9 | transcription factor | 2.1 | NC | 2.4 | 1.7 | 1.6 | NC |
| C3orf58 | chromosome 3 open reading frame 58 | unknown | 2.0 | 1.4 | 2.4 | NC | 2.9 | NC |
| C4orf45 | hypothetical protein | unknown | 1.6 | 1.8 | 2.4 | 1.8 | 1.7 | NC |
| IRS2 | insulin receptor substrate 2 | adaptor for kinase signalling | NC | 1.5 | 2.3 | 1.5 | 1.8 | NC |
| PFKL | phosphofructokinase, liver | glycolysis | NC | NC | 2.3 | 1.7 | 1.6 | NC |
| PGM1 | phosphoglucomutase 1 | glycolysis | 1.8 | 1.3 | 2.3 | 1.7 | 2.2 | NC |
| PLOD2 | procollagen-lysine, 2-oxoglutarate 5-dioxygenase 2 | hydroxylates lysines in collagen | 1.9 | 1.2 | 2.3 | 1.6 | 4.0 | NC |
| DUSP3 | dual specificity phosphatase 3 | inactivates MAP kinases | 1.7 | 1.7 | 2.3 | 1.4 | 1.7 | NC |
| SAV1 | salvador homolog 1 | MST1 binding protein | 1.5 | 1.5 | 2.3 | NC | 2.0 | NC |
| SLC25A36 | solute carrier family 25, member 36 | unknown | 1.6 | 1.5 | 2.3 | NC | 2.1 | NC |
| PGK1P1 | phosphoglycerate kinase 1, pseudogene 1 | pseudogene | 1.7 | NC | 2.3 | 1.7 | 2.4 | NC |
| PTPRB | protein tyrosine phosphatase, receptor type B | cell adhesion, growth | NC | 2.0 | 2.2 | 3.1 | 2.1 | NC |
| SLC2A1 | solute carrier family 2 (Glut 1) | glucose uptake | 1.9 | NC | 2.2 | 1.5 | 2.9 | NC |
| LGALS8 | lectin, galactoside-binding, soluble, 8 (galectin 8) | integrin-like interactions | 1.6 | 1.5 | 2.2 | 1.6 | 1.7 | NC |
| SOD2 | superoxide dismutase 2, mitochondrial | redox control | 1.5 | NC | 2.2 | 1.7 | 1.6 | NC |
| FZD8 | frizzled homolog 8 | Wnt receptor signaling pathway | 1.9 | 1.5 | 2.2 | NC | 2.5 | NC |
| KDM4B | lysine (K)-specific demethylase 4B | histone demethylase | 1.6 | 1.6 | 2.1 | 1.8 | 2.5 | NC |
| PAM | peptidylglycine alpha-amidating monooxygenase | hormone processing | NC | NC | 2.1 | 1.5 | 1.7 | NC |
| NFIL3 | nuclear factor, interleukin 3 regulated | transcription regulation | 1.9 | 1.7 | 2.1 | 1.8 | 2.4 | NC |
| PIGF | phosphatidylinositol glycan, class F | (GPI)-anchor biosynthesis | 1.7 | NC | 2.1 | 1.7 | 1.5 | NC |
| KCNMB4 | potassium large conductance calcium-activated channel, subfamily M, beta member 4 | potassium uptake | NC | NC | 2.0 | 2.1 | 1.6 | NC |
| MEF2A | MADS box transcription enhancer factor 2, polypeptide A | transcription factor | NC | NC | 2.0 | 1.7 | 1.5 | NC |
| ZNF292 | zinc finger protein 292 | transcription factor | 1.9 | 1.6 | 2.0 | 1.5 | 2.5 | NC |
| MOBKL2A | MOB1, Mps One Binder kinase activator-like 2A | unknown | NC | NC | 2.0 | 1.7 | 1.7 | NC |
