# Supplementary material for: Inhibition of Iron Uptake Is Responsible for Differential Sensitivity to V-ATPase Inhibitors in Several Cancer Cell Lines
Source: PLoS One. 2010 Jul 16;5(7):e11629. doi: 10.1371/journal.pone.0011629 (PMC2905441; doi:10.1371/journal.pone.0011629)
Supplement: Table S5 — Genes Increasing Expression Late with V-ATPase Inhibitors and not with DFO or Low LDL. Genes upregulated 2-fold or more after 24 hours in cells treated with V-ATPase inhibitors and in cells treated with 100 µM deferoxamine are listed in order of increase with Baf at 24 hours. Baf, 15 nM bafilomycin A; LX, 200 nM LX1077; DFO, 100 µM deferoxamine; low LDL, cells incubated in medium containing LDL depleted serum. (0.12 MB DOC) [file pone.0011629.s005.doc]

|  |  |  | **Average Fold Increase** | | | | | |
| --- | --- | --- | --- | --- | --- | --- | --- | --- |
| **Gene** | **Name** | **Function or Pathway** | **Baf 12h** | **LX 12h** | **Baf 24h** | **LX 24 h** | **DFO 12h** | **Low LDL 12h** |
| MVD | mevalonate (diphospho) decarboxylase | cholesterol biosynthesis | 10.6 | 5.3 | 26.0 | 13.5 | NC | NC |
| TM7SF2 | delta14-sterol reductase activity | cholesterol biosynthesis | 7.7 | 4.4 | 19.7 | 13.9 | NC | NC |
| LIPG | lipase, endothelial | phospholipase | 5.9 | 3.1 | 11.3 | 3.9 | NC | NC |
| LSS | lanosterol synthase | cholesterol biosynthesis | NC | NC | 8.9 | 2.9 | NC | NC |
| FADS2 | fatty acid desaturase 2 | lipid biosynthesis | 3.4 | 2.4 | 5.5 | 3.5 | NC | NC |
| HIP1R | huntingtin interacting protein 1 related | actin assembly on endocytic membranes | 5.3 | 1.5 | 5.5 | 4.6 | NC | NC |
| WIPI1 | WD repeat domain, phosphoinositide interacting 1 | autophagy | 2.8 | 1.9 | 4.4 | 2.5 | NC | NC |
| SV2A | synaptic vesicle glycoprotein 2A | calcium regulated secretion | NC | NC | 4.4 | 3.4 | NC | NC |
| FADS1 | fatty acid desaturase 1 | lipid biosynthesis | 2.9 | 1.9 | 4.0 | 3.0 | NC | NC |
| TRIB3 | tribbles homolog 3 | putative protein kinase | 2.5 | NC | 4.0 | 1.9 | NC | NC |
| FABP3P2 | fatty acid binding protein 3, pseudogene 2 | unknown | 2.6 | NC | 3.4 | 2.4 | NC | NC |
| FST | follistatin | signal transduction | 2.4 | 7.0 | 3.4 | 1.9 | NC | NC |
| ACLY | ATP citrate lyase | synthesis of cytosolic acetyl-CoA | 1.9 | NC | 3.2 | 2.0 | NC | NC |
| CLEC2B | C-type lectin, superfamily member 2 | carbohydrate recognition, adhesion | 1.9 | 2.1 | 3.2 | 2.1 | NC | NC |
| MAN1A1 | mannosidase, alpha, class 1A, member 1 | Golgi glycosidase | 1.9 | NC | 3.1 | 1.8 | NC | NC |
| CYB5B | cytochrome b5 type B | mitochondrial cytochrome B | 2.1 | 1.5 | 2.8 | 1.9 | NC | NC |
| ETV5 | ets variant gene 5 | transcription factor | 2.9 | NC | 2.8 | 1.9 | NC | NC |
| MCM3 | MCM3 minichromosome maintenance deficient 3 | DNA replication | NC | 1.6 | 2.8 | 1.7 | NC | NC |
| ETV1 | ets variant gene 1 | transcription factor | 2.6 | 1.5 | 2.8 | NC | NC | NC |
| SCML1 | sex comb on midleg-like 1 | unknown | 2.2 | 1.4 | 2.7 | NC | NC | NC |
| YPEL5 | yippee-like 5 | unknown | 2.1 | 1.6 | 2.7 | 1.7 | NC | NC |
| CDT1 | chromatin licensing and DNA replication factor 1 | DNA replication factor | 1.6 | NC | 2.7 | 1.4 | NC | NC |
| WBP2 | WW domain binding protein 2 | unknown | 2.1 | 1.5 | 2.7 | 1.5 | NC | NC |
| COL6A1 | collagen, type VI, alpha 1 | extracellular matrix component | 2.1 | NC | 2.6 | 1.7 | NC | NC |
| SREBF2 | sterol regulatory element binding transcription factor 2 | cholesterol homeostasis | 2.0 | NC | 2.6 | 1.9 | NC | NC |
| OPTN | optineurin | vesicle traffic | 1.6 | 1.5 | 2.6 | 1.8 | NC | NC |
| CENPI | centromere protein I | response to FSH | 1.7 | 1.6 | 2.6 | 1.7 | NC | NC |
| RDH11 | retinol dehydrogenase 11 (all-trans and 9-cis) | retinal reductase | 2.2 | 1.5 | 2.5 | 1.7 | NC | NC |
| PHLDA1 | pleckstrin homology-like domain, family A, member 1 | antiapotpotic | 1.9 | 1.8 | 2.5 | 1.7 | NC | NC |
| TXNDC16 | thioredoxin domain containing 16 | redox homeostasis | 1.5 | NC | 2.5 | 1.9 | NC | NC |
| PDE5A | phosphodiesterase 5A, cGMP-specific | regulation of cyclic nucleotides | NC | NC | 2.5 | 1.7 | NC | NC |
| LIPA | lipase A, lysosomal acid, cholesterol esterase | lipid catabolism | 1.7 | NC | 2.5 | 1.8 | NC | NC |
| ZC3H12C | zinc finger CCCH-type containing 12C | unknown | NC | NC | 2.5 | 1.9 | NC | NC |
| DKK3 | dickkopf homolog 3 | wnt signaling | 1.5 | NC | 2.5 | 1.8 | NC | NC |
| C6orf223 | chromosome 6 open reading frame 223 | unknown | NC | NC | 2.5 | 2.0 | NC | NC |
| WFDC1 | WAP four-disulfide core domain 1 | suggested tumor suppressor | 1.5 | NC | 2.4 | 2.1 | NC | NC |
| DYX1C1 | dyslexia susceptibility 1 candidate 1 | unknown | 1.9 | 1.7 | 2.4 | 1.7 | NC | NC |
| RSHL2 | radial spokehead-like 2 | putative dynein regulator | 1.4 | NC | 2.4 | 1.6 | NC | NC |
| CDT1 | DNA replication factor | cell cyle | NC | NC | 2.4 | 1.3 | NC | NC |
| SLC17A5 | solute carrier family 17 (anion/sugar transporter), member 5 | sialic acid transport | 2.0 | 1.4 | 2.3 | 1.6 | NC | NC |
| CCPG1 | cell cycle progression 1 | unknown | NC | NC | 2.3 | 1.6 | NC | NC |
| GRN | granulin | peptide hormone precursor | NC | NC | 2.3 | 1.9 | NC | NC |
| MOSPD1 | motile sperm domain containing 1 | unknown | 1.6 | 1.7 | 2.2 | 1.6 | NC | NC |
| RRAGC | Ras-related GTP binding C | unknown | 1.6 | 2.1 | 2.2 | 1.6 | NC | NC |
| PPM2C | protein phosphatase 2C, magnesium-dependent, catalytic subunit | may dephosphorylate PDH | 1.7 | 1.6 | 2.2 | NC | NC | NC |
| TMEM55B | transmembrane protein 55B | PIP2 4-phosphatase | 1.6 | 1.5 | 2.2 | 1.7 | NC | NC |
| SLC16A6 | solute carrier family 16 (monocarboxylic acid transporters), member 6 | MCT family member, function unknown | 1.7 | 2.2 | 2.2 | 2.0 | NC | NC |
| NPC2 | Niemann-Pick disease, type C2 | cholesterol transport | NC | 1.4 | 2.2 | 2.1 | NC | NC |
| ABHD3 | abhydrolase domain containing 3 | function unknown | 1.7 | 1.7 | 2.2 | 1.7 | NC | NC |
| PIGB | phosphatidylinositol glycan, class B | GPI-anchor biosynthesis | NC | NC | 2.2 | 1.6 | NC | NC |
| C4orf45 | chromosome 4 open reading frame 45 | unknown | 1.7 | 1.6 | 2.1 | 1.9 | NC | NC |
| OTUD1 | OTU domain containing 1 | deubiquitylating enzyme | 1.5 | NC | 2.1 | 1.7 | NC | NC |
| FOLR1 | folate receptor 1 precursor | folate uptake | NC | 1.7 | 2.1 | 2.1 | NC | NC |
| MXD1 | MAX dimerization protein 1 | transcriptional repressor | 4.1 | NC | 2.1 | 4.1 | NC | NC |
| CETN2 | centrin, EF-hand protein, 2 | structural component of the centrosome | 1.6 | NC | 2.1 | 1.9 | NC | NC |
| YPEL2 | yippee-like 2 (Drosophila) | unknown | 1.7 | 1.7 | 2.0 | 1.6 | NC | NC |
| STS | steroid sulfatase (microsomal), arylsulfatase C, isozyme S | converts sulfated steroid precursors to estrogens | 1.5 | NC | 2.0 | 1.7 | NC | NC |
| GALNT3 | UDP-N-acetyl-alpha-D-galactosamine:polypeptide N-acetylgalactosaminyl transferase 3 | protein glycosylation | 1.6 | NC | 2.0 | 1.5 | NC | NC |
| PBXIP1 | pre-B-cell leukemia transcription factor interacting protein 1 | transcription corepressor | NC | 1.5 | 2.0 | 1.7 | NC | NC |
| SLFN5 | schlafen family member 5 | unknown | 2.0 | 1.8 | 2.0 | 1.7 | NC | NC |
| ECH1 | enoyl Coenzyme A hydratase 1, peroxisomal | beta-oxidation | NC | NC | 2.0 | 1.6 | NC | NC |
| TAPBP | TAP binding protein (tapasin) | petide loading on MHC I | NC | NC | 2.0 | 1.5 | NC | NC |
