# Supplementary material for: Inhibition of Iron Uptake Is Responsible for Differential Sensitivity to V-ATPase Inhibitors in Several Cancer Cell Lines
Source: PLoS One. 2010 Jul 16;5(7):e11629. doi: 10.1371/journal.pone.0011629 (PMC2905441; doi:10.1371/journal.pone.0011629)
Supplement: Table S6 — Pathways significantly enriched in genes changing expression after 24 h treatment with 15 nM bafilomycin. Genes changing significantly in duplicate experiments were analyzed with Ingenuity Pathways Analysis software to identify pathways in which a statistically significant number of genes were changed. Only pathways with a -log p value greater than 2 by Fisher's exact T-test are shown. The “ratio” is the fraction of total genes assigned to the pathway that changed expression. (0.09 MB DOC) [file pone.0011629.s006.doc]

| **Ingenuity Canonical Pathways** | **-log (p value)** | **Ratio** | **Down** | **No change or no overlap** | **Up** |
| --- | --- | --- | --- | --- | --- |
|  |  |  |  |  |  |
| **Cellular stress** |  |  |  |  |  |
| NRF2-mediated Oxidative Stress Response | 6.59 | 0.34 | 27/183 | 121/183 | 35/183 |
| Regulation of eIF4 and p70S6K Signaling | 3.90 | 0.29 | 18/130 | 93/130 | 19/130 |
| EIF2 Signaling | 3.12 | 0.29 | 15/100 | 71/100 | 14/100 |
| HMGB1 Signaling | 2.66 | 0.31 | 7/98 | 68/98 | 23/98 |
|  |  |  |  |  |  |
| **Cell cycle, growth, proliferation** |  |  |  |  |  |
| Protein Ubiquitination Pathway | 6.02 | 0.32 | 56/201 | 136/201 | 9/201 |
| Mitotic Roles of Polo-Like Kinase | 5.89 | 0.45 | 25/62 | 34/62 | 3/62 |
| RAN Signaling | 4.57 | 0.48 | 11/23 | 12/23 | 0/23 |
| IGF-1 Signaling | 4.10 | 0.34 | 10/100 | 66/100 | 24/100 |
| VEGF Signaling | 3.29 | 0.31 | 15/97 | 67/97 | 15/97 |
| mTOR Signaling | 2.87 | 0.27 | 18/156 | 114/156 | 24/156 |
| PI3K/AKT Signaling | 2.72 | 0.27 | 13/137 | 100/137 | 24/137 |
| 14-3-3-mediated Signaling | 2.22 | 0.29 | 11/114 | 81/114 | 22/114 |
| AMPK Signaling | 2.21 | 0.23 | 15/165 | 127/165 | 23/165 |
|  |  |  |  |  |  |
| **Nucleotide metabolism** |  |  |  |  |  |
| Purine Metabolism | 5.36 | 0.21 | 74/439 | 345/439 | 20/439 |
| Pyrimidine Metabolism | 3.90 | 0.22 | 39/231 | 181/231 | 11/231 |
|  |  |  |  |  |  |
| **Lipid metabolism** |  |  |  |  |  |
| Biosynthesis of Steroids | 4.60 | 0.13 | 4/128 | 111/128 | 13/128 |
|  |  |  |  |  |  |
| **Amino acid metabolism** |  |  |  |  |  |
| Valine, Leucine and Isoleucine Degradation | 3.02 | 0.23 | 14/111 | 86/111 | 11/111 |
| Valine, Leucine and Isoleucine Biosynthesis | 2.20 | 0.16 | 5/44 | 37/44 | 2/44 |
| Alanine and Aspartate Metabolism | 2.15 | 0.18 | 9/88 | 72/88 | 7/88 |
| β-alanine Metabolism | 2.00 | 0.18 | 11/98 | 80/98 | 7/98 |
|  |  |  |  |  |  |
| **Cancer** |  |  |  |  |  |
| ERK/MAPK Signaling | 3.00 | 0.27 | 21/192 | 140/192 | 31/192 |
| Prostate Cancer Signaling | 2.69 | 0.28 | 7/96 | 69/96 | 20/96 |
| Glioma Signaling | 2.73 | 0.28 | 13/112 | 81/112 | 18/112 |
| p53 Signaling | 2.20 | 0.30 | 10/92 | 64/92 | 18/92 |
| Breast Cancer Regulation by Stathmin1 | 2.15 | 0.25 | 28/199 | 149/199 | 22/199 |
|  |  |  |  |  |  |
| **Nuclear Receptor** |  |  |  |  |  |
| RAR Activation | 2.91 | 0.27 | 23/181 | 132/181 | 26/181 |
| PPARα/RXRα Activation | 2.42 | 0.26 | 23/182 | 135/182 | 24/182 |
| Aryl Hydrocarbon Receptor Signaling | 2.03 | 0.25 | 21/154 | 116/154 | 17/154 |
| PPAR Signaling | 2.43 | 0.29 | 11/98 | 70/98 | 17/98 |
|  |  |  |  |  |  |
| **Intracellular signaling** |  |  |  |  |  |
| Glucocorticoid Receptor Signaling | 2.82 | 0.25 | 36/280 | 211/280 | 33/280 |
| Protein Kinase A Signaling | 2.79 | 0.25 | 37/315 | 236/315 | 42/315 |
| Insulin Receptor Signaling | 2.52 | 0.28 | 12/140 | 101/140 | 27/140 |
| Phospholipase C Signaling | 2.13 | 0.24 | 20/253 | 193/253 | 40/253 |
|  |  |  |  |  |  |
| **Carbohydrate metabolism** |  |  |  |  |  |
| Citrate Cycle | 2.59 | 0.22 | 8/58 | 45/58 | 5/58 |
| Fructose and Mannose Metabolism | 2.52 | 0.15 | 6/145 | 124/145 | 15/145 |
| Propanoate Metabolism | 2.22 | 0.17 | 11/130 | 108/130 | 11/130 |
|  |  |  |  |  |  |
| **Disease specific pathways** |  |  |  |  |  |
| Role of NFAT in Cardiac Hypertrophy | 2.76 | 0.25 | 19/207 | 155/207 | 33/207 |
| Mitochondrial Dysfunction | 2.27 | 0.22 | 33/171 | 133/171 | 5/171 |
|  |  |  |  |  |  |
| **Cytokine** |  |  |  |  |  |
| fMLP Signaling in Neutrophils | 2.28 | 0.26 | 14/125 | 93/125 | 18/125 |
|  |  |  |  |  |  |
| **Neurotransmiter function** |  |  |  |  |  |
| Cholecystokinin/Gastrin-mediated Signaling | 2.42 | 0.30 | 9/104 | 73/104 | 22/104 |
| Agrin Interactions at Neuromuscular Junction | 2.05 | 0.32 | 9/69 | 47/69 | 13/69 |
